# Knowledge, attitudes and practices of dengue prevention between dengue sustained hotspots and non-sustained hotspots in Singapore: a cross-sectional study

**DOI:** 10.1038/s41598-022-22776-y

**Published:** 2022-11-01

**Authors:** Jeth WeiQuan Chng, Tamilsalvan Parvathi, Junxiong Pang

**Affiliations:** 1 Saw Swee Hock School of Public Health, Tahir Foundation Building, National University of Singapore, 12 Science Drive 2, #10-01, Singapore, 117549 Singapore; 2 Western Regional Office, Environmental Public Health Operations Group, National Environment Agency, Singapore, Singapore

**Keywords:** Risk factors, Infectious diseases, Epidemiology

## Abstract

Dengue sustained hotspots (SHS) have resulted in a significant public health burden. In our study, we aimed to (1) compare knowledge, attitudes and practices (KAP) scores between SHS and non-sustained hotspots (NSHS); and (2) identify and describe gaps and factors associated with KAP of dengue prevention among SHS residents residing in Singapore. A cross-sectional study with convenience sampling was conducted using digital survey in randomly selected SHS and NSHS residential areas, consisting of residents aged 21 or older and who had been residing in their existing housing unit in 2019 and 2020. Chi-square test and T-test were used for comparison analysis of categorical and continuous variables, respectively. A total of 466 respondents completed the self-administered, anonymous survey. There were no significant difference in mean scores for Knowledge [SHS(24.66) vs. NSHS(24.37); P: 0.18], Attitudes [SHS(10.38) vs NSHS(10.16); P: 0.08] and Practices [SHS(9.27) vs NSHS(8.80); P: 0.16] sections. Significant SHS-associated factors identified were age group 41–50 years old [95%CI: 1.25–5.03], Malay (95%CI: 0.17–0.98), up to secondary school education (95%CI: 0.07–0.65), private condominium (95%CI: 1.17–3.39), residing in same household unit for 2–5 years (95%CI: 2.44–6.88), respondents who know that mosquito can breed in open container with stagnant water (95%CI: 0.06–0.98), disagree that reducing *Aedes* mosquitoes is the only way to prevent dengue: (95%CI: 1.19–3.00) and go to clinic/hospital even without severe symptoms: (95%CI: 0.39–0.95). These independent factors associated with dengue sustained hotspots may influence the risk of dengue transmission in residential areas.

## Introduction

Dengue Fever (DF) is a viral infection caused by the four serotypes of dengue virus and transmitted by the *Aedes* mosquito^1^. In the past two decades, global incidence of dengue has increased more than ten-fold, from 505,430 cases in 2000 to over 5.2 million cases in 2019^1^. In 2013, an estimated US$ 8.9 billion was incurred globally due to dengue illness among symptomatic cases, with more than half the world’s population predicted to be at risk of dengue illness annually using modelling frameworks^2,3^. DF remains the most common mosquito-borne fever in South-east Asia^4^. Locally, Singapore experienced its highest number of dengue cases in 2020, with a total of 35,315 cases, and 32 reported deaths^5^.

While vector control remains the primary method of mitigating dengue cases and clusters in Singapore, several key factors have fueled the rise in DF in recent years and limited the effectiveness of local vector control methods^1,6,7^. These factors include an increasingly urbanized environment suitable for thriving *Aedes* mosquito propagation, the expansion of population with intra-urban human movement, a delay in public participation only after dengue cluster emerges, the difficulty of sustaining year-long anti-dengue campaign, as well as the lack of clinical manifestation for asymptomatic cases^7–11^. These factors, which are prevalent to Singapore, meant that the country is highly susceptible to dengue clusters, and the national disease burden remains high.

In Singapore, dengue cluster is defined by the National Environment Agency (NEA), a government statutory board in Singapore responsible for local public health, as two or more dengue cases occurring within 14 days, with residential address of the dengue cases being within 150 m of each other^12^. NEA is tasked with sustaining local vector control efforts to curb dengue, and the above-mentioned key factors may have contributed to the propensity of prolonged or repeated dengue clusters. Modelling after practice guidelines from the World Health Organization (WHO), a combination of *Aedes* mosquito control measures to combat DF was implemented in Singapore’s context, which included source reduction, active dengue surveillance, regulations, enforcement and public education as part of its national strategy^11,13^. Recent measures implemented include the islandwide deployment of Gravitraps (local dengue control tool used to attract and trap female *Aedes* mosquitoes), phased introduction of Project *Wolbachia* (mosquito suppression tool utilizing *Wolbachia* bacteria to fight dengue), as well as publicity campaigns through Dengue Prevention Volunteers and the National Dengue Prevention Campaign^14,15^. As part of its public education programme, NEA engages the community through community roadshows and educational materials (online and leaflets)^16^. In addition, the advent of open data available to the public is also evident in the impact of dengue control in Singapore. Daily updates on dengue cluster information in NEA’s website increases public awareness on the severity of dengue and prevention measures to be taken, while the public is also encouraged to report on potential mosquito breeding sites and adult mosquito nuisance through NEA’s feedback system, to encourage active public participation in curbing dengue threats in Singapore^17,18^.

To evaluate the impact and success of these measures, the Knowledge, Attitudes and Practices (KAP) survey model serves as a useful assessment tool to evaluate the readiness of the population in responding to dengue outbreaks^19,20^. Studies in other countries showed that public knowledge on dengue is necessary to mitigate the spread of the virus, and increasing dengue knowledge helped to control dengue outbreak^21,22^. At present, there are limited overseas studies comparing dengue KAP of residents in dengue hotspots only. Although the studies varied in community dengue-related knowledge levels, they concluded that higher dengue knowledge within the community was essential to sustain dengue preventive efforts^23–25^. In a local study on household mosquito breeding measures in 2010, it was revealed that residents in dengue hotspots were generally more knowledgeable compared to their non-hotspot counterparts^26^. However, the paper revealed a knowledge-practice gap, which the respondents were not compliant with mosquito breeding prevention measures despite their knowledge on the subject matter. The limited KAP studies focusing on hotspots suggested a lack of understanding of residents’ KAP levels living in hotspots. This in turn was crucial in identifying KAP gaps, assessing current public vector control strategies and developing targeted interventions for high-risk groups. Dengue KAP study could potentially play an essential role in unravelling key determinants resulting in lack of KAP within the community, which can be mitigated through targeted policies and initiatives aimed at curbing the growing incidence of dengue clusters and reducing the spread of sustained hotspots, both in the global and local settings.

This study aimed to compare dengue KAP among residents in dengue sustained hotspots (SHS) and non-sustained hotspots (NSHS) areas and to evaluate the existence of knowledge-attitudes gap and/or knowledge-practices gap, and identify risk factors associated with sustained hotspots, if any. These risk factors may provide more targeted evidence-based approach to reduce the risk of dengue transmission in dengue sustained hotspots.

## Methods

### Study design and population

This was a community-based, cross-sectional study, that was conducted between April 2021 to July 2021. The targeted population consisted of local residential households located in both dengue SHS and NSHS areas in Singapore. NEA defines dengue cluster as a locality with active dengue transmission when two or more cases have onset within 14 days and are located within 150 m within each other (based on residential address for our study)^17^. We defined “sustained hotspot” as an area with at least 2 dengue clusters within a two year period (i.e. period of 2019 to 2020)^26^. NSHS was defined as an area with one or no dengue clusters in the same period. Inclusion criteria were participants aged 21 years old and above from all nationalities, and they had been staying in their existing housing unit for the past 2 years (2019–2020). Exclusion criteria applied to participants who resided less than 2 years in total, stayed in the household less than 3 days in a week and aged younger than 21 years old during the date of survey done.

### Study instruments and data collection

The questionnaire was developed using Research Electronic Data Capture (REDCap™), and the reliability and validity of the questionnaire was checked by pre-testing it with 25 respondents. Using convenience sampling, the questionnaire was self-administered through a survey link, which took approximately 10 min to complete. Translation of the other local languages (Mandarin, Malay and Tamil) was carried out, so as to enable the survey to reach out to a wider range of respondents. The survey link was then shared via Facebook advertisement, WhatsApp, and targeted flyer distribution. While survey was conducted anonymously, residential postal code was the only identifier requested to enable the matching of NEA’s records to allocate the respondent into SHS or NSHS category, as information on hotspot areas was not publicly available. Our study respondents resided in various housing types such as Housing Development Board (HDB)’s public owned units in blocks, public rental units in blocks, private owned landed properties as well as private owned units within condominiums.

The 89-item KAP online questionnaire (Supplementary Figs. 1–4) consisted of 4 sections in this order: 25 questions on demographic characteristics, 33 questions on knowledge of dengue symptoms and mosquito preventive measures; 14 questions on attitudes towards transmission, prevention and diagnosis; and 17 questions on practices such as the use of mosquito repellent. Respondents who selected “I agree” at the consent for participation of study page were directed to complete the 4 sections. Respondents who selected “I disagree” at the consent for participation of study page were routed to a one-page section consisting of 7 questions on personal and socio-demographic characteristic, to obtain their baseline characteristics for reference.

For data collection, only inputs of respondents who indicated a valid postal code were processed. The responses from each section of KAP were measured using a 7-point Likert scale for level of agreement, with options such as “Strongly Disagree”, “Disagree”, “Somewhat Disagree”, “Neutral”, “Somewhat Agree”, “Agree” and “Strongly Agree”. A correct or positive response, such as “Somewhat Agree”, “Agree” and “Strongly Agree” would constitute 1 point for each question, while respondents who chose the other options would be scored 0 points. In addition, a 7-point Likert scale for frequency is also employed in the “Practices” section, to identify how often respondents engage in the activity. The options include “Never”, “Once a week”, “2 times a week”, “3 times a week”, “4 times a week”, “5–6 times a week” and “Daily”, with 1 point scored for any choice of option that is defined as at least 3 times a week. The majority of questions were multiple choice, with the exception of several questions which included an option for “Others”. This had free text enabled for respondents to indicate their unique responses. For the KAP sections, 1 point was scored for each correct answer or positive behaviour. Positive or correct answers for the KAP sections were summed, with the total score of each section being obtained for each respondent.

### Statistical analysis

Information captured by REDCap™ was transferred to an Excel database, where entered data were used to perform initial descriptive analysis. The data were then imported into Stata version 13.1.226 for both descriptive and comparative analysis. Data were checked twice to ensure replicability and accuracy. Descriptive statistics were conducted to obtain frequencies and percentages. Categorical variables for socio-demographic characteristics was interpreted and compared using Chi square test, while t-test was used to compare the mean of the KAP score between the two study groups. Multivariable logistic regression model for sustained hotspots was developed through regression analysis of significant variables from the socio-demographic section using Likelihood Ratio Test and Goodness-of-Fit test. Forward stepwise regression was conducted for all sociodemographic variables, to assess significance of the variables to be included into null model, to obtain ‘best’ logistic regression model. Likelihood Ratio Test and Pearson’s Goodness-of-Fit test were conducted to ensure parsimonious model is used. The logistic regression analysis results were reported as Crude Odds Ratio (COR) and Adjusted Odds Ratio (AOR), with their corresponding 95% Confidence Intervals (CI). Cross-tabulations were calculated for SHS, NSHS and ALL (SHS + NHS) groups, i.e. between knowledge and practices, knowledge and attitudes, and attitudes and practices, using pairwise correlation table. Cronbach’s alpha reliability testing and Skewness/Kurtosis tests for Normality were also conducted (Supplementary Tables 1 and 2). Statistical significance was set at *P* < 0.05.

### Ethical consideration

The study protocol was approved by the National University of Singapore (NUS) Institutional Review Board (IRB) NUS-IRB (Reference Code: NUS-IRB-2020-618). All methods were performed in accordance with the relevant guidelines and regulations. Online informed consent was obtained at start of the questionnaire for those who agreed to participate in study. Waiver of written informed consent was approved by NUS IRB since there are no personal identifiers collected and the survey was performed anonymously.

## Results

A total of 515 individual assessments were collected, with 466 respondents completing (n = 346 for SHS, n = 120 for NSHS) and 49 respondents partially completing the questionnaire, with a 90.5% completion rate. Among SHS respondents, 266 resided in public HDB and rental blocks (77%), 51 lived in Condominium (15%) and 29 stayed in landed properties (8%). As for NSHS respondents, 72 resided in HDB and rental blocks (60%), 42 lived in Condominiums (35%) and 6 stayed in landed properties (5%). Using Chi square test, there were statistically significant differences observed in age [*P* < 0.01], educational level [*P* = 0.01], marital status [*P* < 0.01] housing type [*P* < 0.01], years staying in house [*P* < 0.01], gross monthly income [*P* = 0.01], hours at home in 2020 on weekends [*P* = 0.03] and hours of air-conditioning use on weekends [*P* = 0.03] between SHS and NSHS areas (Table 1 and Supplementary Table 3).

**Table 1:** Demographic and significant characteristic differences between SHS and NSHS.

Demographic characteristic		Respondents (No. %)			χ2 /T- Test	Crude odds ratio (COR)			Adjusted odds ratio (AOR)*		
		All	SHS	NSHS	*P*-value	OR	95% CI	*P*-value	OR	95% CI	*P*-value
Gender	Female	249 (53)	189 (55)	60 (50)	**–**	1.00	–	–	1.00	–	–
	Male	217 (47)	157 (45)	60 (50)	0.38	1.20	0.79–1.82	0.38	1.33	0.85–2.08	0.22
Age	Mean^	41.2	41.0	41.7	0.60	1.00	0.98–1.02	0.87	1.01	0.95–1.07	0.86
	31–40 years old		121 (35)	43 (36)	** < 0.01**	1.00	–	–	1.00	–	–
	21–30 years old		81 (23)	17 (14)		1.69	0.90–3.17	0.10	1.23	0.64–2.38	0.54
	41–50 years old		57 (17)	36 (30)		3.01	1.54–5.87	** < 0.01**	2.50	1.25–5.03	**0.01**
	> 50 years old		87 (25)	24 (20)		1.31	0.66–2.62	0.44	1.78	0.86–3.70	0.12
Ethnicity	Chinese	324 (70)	237 (68)	87 (72)	0.09	1.00	–		1.00	–	–
	Malay	52 (11)	45 (13)	7 (6)		0.42	0.18–0.98	**0.043**	0.41	0.17–0.98	**0.04**
	Indians and others	90 (19)	64 (19)	26 (22)		1.11	0.66–1.86	0.70	0.87	0.50–1.54	0.64
Educational level	Post-Secondary	423 (91)	307 (89)	116 (97)	**0.01**	1.00	–	–	1.00	–	–
	Up to Secondary school	43 (9)	39 (11)	4 (3)		0.27	0.09–0.78	**0.015**	0.22	0.07–0.65	** < 0.01**
Marital Status	Married	240 (51)	159 (46)	81 (67)	** < 0.01**	1.00	–		1.00	–	–
	Single	213 (46)	177 (51)	36 (30)		0.40	0.26–0.62	** < 0.01**	0.59	0.33–1.03	0.06
	Divorcee/Widow/ Widower	13 (3)	10 (3)	3 (3)		0.59	0.16–2.20	0.43	1.37	0.31–5.94	0.68
Employment Status	Working	375 (80)	282 (81)	93 (77)	0.34	1.00	–		1.00	–	–
	Non-working	91 (20)	64 (19)	27 (23)		1.28	0.77–2.12	0.34	1.31	0.76–2.28	0.33
Housing Type	HDB & Rental Blocks	338 (73)	266 (77)	72 (60)	** < 0.01**	1.00	–		1.00	–	–
	Condominium	93 (20)	51 (15)	42 (35)		3.04	1.87–4.94	** < 0.01**	1.99	1.17–3.39	**0.01**
	Landed	35 (7)	29 (8)	6 (5)		0.76	0.31 –1.91	0.57	0.64	0.24–1.67	0.36
Years staying in house	More than 10	237 (51)	202 (59)	35 (29)	** < 0.01**	1.00	–		1.00	–	–
	5 to 10	83 (18)	57 (16)	26 (22)		2.63	1.46–4.73	** < 0.01**	2.60	1.42–4.75	** < 0.01**
	2 to 5	146 (31)	87 (25)	59 (49)		3.91	2.40–6.38	** < 0.01**	4.10	2.44–6.88	** < 0.01**
Gross monthly income ($)	> 7,000	231 (49)	158 (46)	73 (61)	**0.01**	1.00	–	–	1.00	–	–
	3,000–6,999	161 (35)	127 (37)	34 (28)		0.58	0.36–0.93	**0.02**	0.83	0.50–1.38	0.48
	< 3,000	74 (16)	61 (17)	13 (11)		0.46	0.24–0.89	**0.02**	0.76	0.36–1.57	0.46
People staying in house	3–4 people	245 (53)	186 (54)	59 (49)	0.05	1.00	–	–	1.00	–	–
	5 or more	128 (27)	100 (29)	28 (23)		0.88	0.53–1.47	0.63	0.72	0.42–1.25	0.25
	1–2 people	93 (20)	60 (17)	33 (28)		1.73	1.04–2.90	**0.037**	1.31	0.75–2.29	0.35
Hours at home (2020, weekends)	Mean^	17.5	17.2	18.3	**0.03**	1.05	1.01–1.10	**0.03**	1.04	0.99–1.10	0.09
	> 12 h	382 (82)	279 (81)	103 (86)		1.00	–	–	1.00	–	–
	≤ 12 h	84 (18)	67 (19)	17 (14)		0.69	0.39–1.23	0.20	0.75	0.41–1.39	0.37
Hours air conditioning (weekends)	Mean^	6.2	5.9	7.2	**0.03**	1.04	1.00–1.08	**0.03**	1.01	0.97–1.06	0.48
	≤ 12 h	419 (90)	317 (92)	102 (85)		1.00	–	**–**	1.00	–	–
	> 12 h	47 (10)	29 (8)	18 (15)		1.93	1.03–3.62	**0.04**	1.36	0.70–2.67	0.36

*Adjusted for Age, Education Level and Years staying in home.

^T-test used. Significant values are in bold.

### Significant factors associated with sustained hotspots (SHS)

Significant factors associated with SHS derived within SHS areas were age [41–50 years old: SHS (17%) vs NSHS (30%); 95% CI, 1.25–5.03], ethnicity [Malay: SHS (13%) vs NSHS (6%); 95% CI, 0.17–0.98], educational level [Up to secondary school: SHS (11%) vs NSHS (3%); 95% CI, 0.07–0.65], housing type [Condominium: SHS (15%) vs NSHS (35%); 95% CI, 1.17–3.39] and years staying in house [2 to 5 years: SHS (25%) vs NSHS (49%); 95% CI, 2.44–6.88 & 5 to 10 years: SHS (16%) vs NSHS (22%); 95% CI, 1.42–4.75] (Table 1 and Supplementary Table 3).

### Knowledge section

For knowledge, there were three associated knowledge factors involved among those who responded correctly to these: “Eggs of mosquitoes can survive up to 6 months in dry conditions” [95% CI 0.41–0.99], “Distance of Flight of *Aedes* mosquitoes is 150 m” [95% CI 0.39–0.98] and “Mosquito can breed in open container with stagnant water” [95% CI 0.06–0.98] (Table 2 and Supplementary Table 4). SHS respondents had similar knowledge levels as NSHS respondents, with no significant difference in mean scores for combined knowledge section (*P* = 0.18) (Supplementary Table 5).

**Table 2:** Comparison of Knowledge topics between SHS (n = 346) and NSHS (n = 120) respondents (significant).

Knowledge characteristic^	Correct response, No. (%)			χ2 test	Crude odds ratio (COR)			Adjusted odds ratio (AOR)*		
	Total	SHS	NSHS	*P*-value	OR	95% CI	*P*-value	OR	95% CI	*P*-value
**Mosquitoes**										
Eggs of mosquitoes can survive up to 6 months in dry conditions	252 (54)	197 (57)	55 (46)	**0.04**	0.64	0.42–0.97	**0.04**	0.64	0.41–0.99	**0.046**
Distance of flight of Aedes mosquitoes is 150 m	210 (45)	166 (48)	44 (37)	**0.03**	0.63	0.41–0.96	**0.03**	0.62	0.39–0.98	**0.041**
**Mosquito Breeding Area**										
Open container with stagnant water	454 (97)	339 (98)	115 (96)	0.20	0.47	0.15–1.53	0.21	0.25	0.06–0.98	**0.046**
**Prevent dengue**										
Using mosquito nets	434 (93)	327 (95)	107 (89)	**0.046**	0.48	0.23–1.00	0.05	0.57	0.25–1.29	0.18
Using mosquito repellent coil	410 (88)	312 (90)	98 (82)	**0.01**	0.49	0.27–0.87	0.02	0.56	0.30–1.04	0.07

*Adjusted for Age, Education Level and Years staying in home.

^Correct response gives 1 point; Wrong or Neutral response give 0 points. Significant values are in bold.

### Attitudes section

Three significant factors associated with SHS involved respondents who agreed on the following perceptions: “I am responsible to keep my place free from mosquito breeding sites” [95% CI 0.13–0.99], “Does not agree that reducing *Aedes* mosquitoes is the only way to prevent dengue” [95% CI 1.19–3.00] and “If I have dengue symptoms, I will seek immediate treatment” [95% CI 0.18–0.84] (Table 3 and Supplementary Table 6). SHS respondents had similar attitudes levels as NSHS respondents, with no significant difference in mean scores for combined attitudes section. (*P* = 0.08) (Supplementary Table 5).

**Table 3:** Comparison of Attitude topics between SHS (n = 346) and NSHS (n = 120) respondents (significant).

Attitudes characteristic^	Positive response No. (%)			χ2 test	Crude odds ratio (COR)			Adjusted odds ratio (AOR)*		
	All	SHS	NSHS	*P*-value	OR	95% CI	*P*-value	OR	95% CI	*P*-value
**How do you feel about the following statements?**										
I am responsible to keep my place free of mosquito breeding sites	446 (96)	334 (97)	112 (93)	0.14	0.50	0.20–1.26	0.14	0.37	0.13–0.99	**0.049**
Reducing Aedes mosquitoes is the only way to prevent dengue	216 (46)	173 (50)	43 (36)	** < 0.01**	0.56	0.36–0.86	** < 0.01**	0.53	0.33–0.84	** < 0.01**
If I have dengue symptoms, I will seek immediate treatment	430 (92)	325 (94)	105 (88)	**0.02**	0.45	0.23–0.91	**0.03**	0.39	0.18–0.84	**0.02**

*Adjusted for Age, Education Level and Years staying in home.

^Positive response gives 1 point; Negative or Neutral response give 0 points. Significant values are in bold.

### Practices section

For “Practices” section, there were two significant factors associated with SHS among residents who practised the following: “Go to clinic / hospital only if symptoms worsen” [95% CI 0.39–0.95] and “Communicating with Dengue volunteers or NEA officers to learn more about dengue” [95% CI 0.32–0.88]. (Table 4 and Supplementary Table 7). SHS respondents had similar practice levels as NSHS respondents, with no significant difference in mean scores for combined practices section. (*P* = 0.16) (Supplementary Table 5).

**Table 4:** Comparison of Practice topics between SHS (n = 346) and NSHS (n = 120) respondents (significant).

Practices characteristic	Positive behaviour /Correct Response No. (%)			χ2 test	Crude odds ratio (COR)			Adjusted odds ratio (AOR)*		
	All	SHS	NSHS	*P*-value	OR	95% CI	*P*-value	OR	95% CI	*P*-value
**Agreement^**										
Go to clinic / hospital only if symptoms worsen	250 (54)	194 (56)	56 (47)	0.08	0.69	0.45–1.04	0.08	0.61	0.39–0.95	**0.03**
Communicating with Dengue volunteers or NEA officers to learn more about dengue	180 (39)	146 (42)	34 (28)	** < 0.01**	0.54	0.35–0.85	** < 0.01**	0.53	0.32–0.88	**0.01**

*Adjusted for Age, Education Level and Years staying in home.

^Correct score gives 1 point; Incorrect or Neutral response give 0 points. Significant values are in bold.

### Mean scores of KAP

There were no statistical significance observed in the mean scores of KAP between SHS and NHS respondents (*P* > 0.05) (Table 5). Pairwise correlation was also conducted for KAP between SHS and NSHS respondents, with no significant association was observed between knowledge and practices (Table 6). However, there was significant positive correlation observed, albeit a low correlation, between knowledge and attitudes scores for NHS (r = 0.20; *P* = 0.03) and SHS (r = 0.14; *P* = 0.01) groups. In addition, there was significant positive correlation observed, albeit a low correlation, between attitudes and practices for NHS group (r = 0.25; *P* < 0.01) (Table 6)^27^.

**Table 5:** Comparison of Mean Scores of KAP between SHS (n = 346) and NSHS (n = 120) respondents.

Characteristic^	Mean score		T-test	Crude odds ratio (COR)			Adjusted odds ratio (AOR)*		
	SHS	NSHS	*P*-value	OR	95% CI	*P*-value	OR	95% CI	*P*-value
Knowledge	24.66	24.37	0.41	0.97	0.91–1.04	0.41	0.95	0.89–1.02	0.18
Attitudes	10.38	10.16	0.28	0.94	0.84–1.05	0.28	0.90	0.79–1.01	0.08
Practices	9.27	8.80	0.22	0.96	0.91–1.02	0.22	0.95	0.89–1.02	0.16

^17 counts from Knowledge, 7 counts from Attitudes and 0 count from Practices removed as outliers (> 1.5 interquartile range).

*Adjusted for Age, Education Level and Years staying in home.

**Table 6:** Pairwise Correlation Matrix for KAP between SHS and NSHS respondents.

KAP–area R (sig lvl)*	K-NSHS	K-SHS	K-ALL	A-NSHS	A-SHS	A-ALL	P-NSHS	PSHS	P-ALL
K-NSHS	–	–	–	0.20 (**0.03**)*	–	–	0.16	–	–
K-SHS	–	–	–	–	0.14 (**0.01**)*	–	–	< 0.01	–
K-ALL	–	–	–	–		0.07	–	–	−0.03
A-NSHS	0.20 (**0.03**)*	–	–	–	–	–	0.25 (**0.01**)*	–	–
A-SHS	–	0.14 (**0.01**)*	–	–	–	–	–	0.02	–
A-ALL	–	–	0.07	–	–	–	–	–	< −0.01
P-NSHS	0.16	–	–	0.25 (**0.01**)*	–	–	–	–	–
P-SHS	–	< 0.01	–	–	0.02	–	–	–	–
P-ALL	–	–	–0.03	–	–	< −0.01	–	–	–

r = Pearson Correlation Coefficient, indicates how far away all these data points are to this line of best fit. Significance at P < 0.05.

Size of correlation interpretation: 0–0.3 (negligible), 0.3–0.5 (low), 0.5–0.7 (moderate), 0.7–0.9 (high), 0.9–1.0 (very high).

Significant values are in bold.

## Discussion

This study explored the knowledge, attitudes and practices (KAP) of residents with regards to dengue prevention, and aimed to elucidate the differences in KAP, if any, between respondents from sustained hotspot (SHS) and non-hotspot (NSHS) in Singapore. The results revealed that there were no statistical differences in overall levels of knowledge, attitudes and practices among both groups. Sociodemographic parameters found to influence the risk of sustained hotspot among residents included age, educational level and years of residence in house. Significant factors associated with SHS identified were summarised in Supplementary Table 6.

Residents aged 41–50 years old was observed to be a significant factor associated with SHS, with 2.5 times higher risk of associated with sustained hotspots, compared to reference age group 21–30 years old. This age group was also associated with increased dengue seroprevalence, dengue incidence and inapparent dengue in Singapore^28–30^. Residing within SHS area, as well as the potential decline in pre-existing antibodies after previous dengue infection, meant that residents aged 41–50 years old constantly faced increased dengue risk especially in the face of prolonged dengue cluster periods^31,32^. Declining antibodies from dengue serotypes was correlated with an increase in DF probability^33^. Age-specific targeted intervention was studied by Sanofi in the application of dengue vaccine, and while there is currently no local study on age-specific public health education intervention on dengue, future interventions on our study findings may unravel potential benefits of age-specific dengue vaccination or other targeted intervention^34^.

Another significant factor associated with SHS was identified as the duration of current residence, i.e. 10 years or less. Residents who have current residence duration for 5–10 years had 2.60 times higher risk of associating with sustained hotspots as compared to residents residing in homes for longer duration (reference group: > 10 years) (Supplementary Table 8). An increasing risk effect of 4.10 times was seen among residents with current residence duration of 2–5 years. In Singapore, new housing units are spaced in closer proximity, with living density in Singapore projected to increase from 11,000 people/km^2^ in 2018 to 13,700 people/km^2^ in 2030^35^. Between 2010 to 2016, 178,000 units of Build-to-Order (BTO) public high-rise housings were supplied, and that these units were built on both newer towns and existing mature estates^36^. Close proximity of housing units was significantly correlated with mosquito abundance and dengue incidence^37–39^. We also hypothesized that these residents may potentially lack immunity against existing circulating serotypes at these selected hotspots, and/ or may not possess sufficient knowledge of residing in hotspot areas. This may result in the lack of compliance and motivation to practise dengue prevention, thus increasing their risk being in SHS. Possible solutions for this group of residents may include the provision of dengue-related information such as prevailing dengue serotype as well as visual aids on potential mosquito breeding areas, which may increase dengue awareness and promote dengue prevention practices.

Residents residing in private condominiums was identified as another significant factor associated with SHS, with a 1.99 times higher risk as compared to residents residing in HDB group. A retrospective exploratory study on prolonged dengue cluster at a high-rise apartment was conducted in Malaysia, which revealed poor community participation and lack of resident cooperation during health education and outreach attempts^40^. The study had recommended for apartment staff to conduct innovative community outreach together with integrated preventive measures to enable better dengue prevention for future outbreaks. While apartment and condominium differ in ownership of unit, the physical attributes and accompanying amenities are similar, whereby a team of staff oversees the maintenance and assistance rendered to residents. The Malaysian study provided a useful reference for modelling local outreach efforts tailored to condominiums in Singapore, and the cooperation between staff and residents would be crucial to mitigate dengue risk in local condominiums.

In the “Knowledge” section, the significant statements associated with SHS factors within “mosquitoes” sub-section could be attributed to weaker general understanding on knowledge involving mosquitoes compared to dengue for SHS respondents. In areas prone to dengue clusters, national public health officers conduct extensive dengue outreach to educate residents on how dengue is transmitted, prevention of mosquito breeding, information on dengue clusters and other frequently asked questions (FAQs)^16^. Specific knowledge involving mosquitoes, such as distance of adult flight and survival of eggs, may not be apparent to residents as this information is usually communicated only if residents specifically asked national public health officers on this. Studies have shown that residents equipped with sufficient mosquito knowledge was essential to effective and timely vector prevention and control programmes^41–43^. Dengue outreach could be modified to include mosquito facts, to promote awareness and adherence to proper mosquito preventive measures. An interesting observation was that “Open container with stagnant water” was also a significantly associated knowledge in SHS areas, whereby use of clear and specific description of mosquito breeding sites could address the knowledge misconceptions among SHS residents.

Within the “Attitudes” section, it was observed that SHS residents may not fully agree that it was their own responsibility to keep their place free from mosquito breeding sites. In Asia, intervention studies have been suggested improving the attitudes of the locals towards DF^44–46^. Locally, while national authority for environment has been responsible with maintaining an effective dengue prevention programme, more could be done to engage residents on maintaining self-responsibility towards dengue prevention, with a national online feedback platform provided for concerned residents to provide their observations of potential mosquitoes breeding sites. Other studies have suggested that partnering with local grassroot leaders to foster residents’ participation has been shown to promote community participation and contribute to a holistic and effective dengue prevention campaign^16,47,48^.

From the ”Practices” section, one important takeaway would be the reluctance of residents’ in engagement with Dengue volunteers and the national public health officers. This could be attributed to the strict enforcement regime imposed on residents who commit mosquito breeding offences within their premise, though effective, might deter past offenders from engaging with dengue officers and volunteers. Other than meeting face-to-face through house visits or dengue flyers, residents might be unaware of the efforts conducted by local dengue volunteers. In Brisbane, the combination of surveillance and regulatory enforcement (warning to residents to spruce housekeeping), was successful in eliminating dengue^49^. However in Singapore’s context, the endemic nature of dengue, coupled with the declining immunity levels of the general population, meant that both public education and enforcement are still required as part of its integrated vector control management to manage dengue^31^. In light of the current covid-19 situation, as well as the aftermath of the 2020 dengue outbreak, public engagement strategies could be re-modelled to keep up with the new circumstances. Besides the traditional house visits and dengue flyers, engaging stakeholders through social media platforms might become the new norm, such as national live Facebook updates during the Zika outbreak in 2016^50^.

Reacting to dengue symptoms was a common theme and significant factor associated with SHS seen in both “Attitudes” and “Practices”, although SHS and NSHS respondents had similar scores with regards to knowledge on dengue symptoms (Tables 2, 3 and 4). Studies in other countries revealed that poor understanding of dengue symptoms was correlated with weaker dengue prevention and control in the area^25,51,52^. From our initial study findings, further KAP study on dengue symptoms could be conducted to elucidate the level of understanding among residents and identify current mindset and behaviour. To ensure residents are updated on knowledge of dengue symptoms, grassroot leaders partnering dengue volunteers could clarify concerns and correct misconceptions among residents, to cultivate positive health-seeking attitudes and behaviour when faced with dengue symptoms.

### Limitations

Our study only covered one aspect of susceptible population, i.e. residents’ KAP. Referring to the epidemiologic triad, dengue disease occurrence results from interaction between the dengue virus agent and the susceptible population within an environment with sufficient abundance of mosquitoes to transmit the virus agent transmitting from source to host. Findings from KAP study alone would not be conclusive in preventing dengue, but would complement existing vector control measures. Understanding the correlation of agent and environment with KAP could provide valuable insights on why an area is a sustained dengue hotspot.

This was a cross-sectional study design, and the observed associations might be due to bias, as well as potential unknown confounders unaccounted for in this analysis, which would affect the internal validity of the study. Being an inexpensive and quick way to conduct the study, convenience sampling was conducted instead of the use of trained interviewers. The digital route of survey distribution eliminated interviewer bias. However, several forms of selection bias were considered. As survey was distributed in randomly selected SHS and NSHS residential areas, we received many more SHS responses than NSHS responses, with Cronbach’s alpha score of 0.43 depicting a lower score for reliability testing (Supplementary Table 1). Furthermore, normality testing had shown that sample skewness and sample kurtosis are necessarily bounded by functions of sample size, imparting bias to the extent that small samples from skewed distributions may even deny their own parentage (Supplementary Table 2)^53^. The possible inadequate representation of NSHS respondents was mitigated using flyer distribution which was targeted at NSHS areas to increase NSHS response. It was also noted in national records that there were more postal codes in our defined SHS areas than NSHS areas (no other information could be revealed), thus consistent in the distribution of respondents in SHS (n = 346) and NSHS (n = 120) areas. A 2019 survey revealed that almost 89% of individuals residing in Singapore used the Internet, but only 58% of residents above 60 years old had internet access^54–56^. Respondents lacking an internet device would be unable to assess the survey, and thus excluded from participating in the survey.

Non-respondent bias might occur as only those concerned, health-conscious or aware on the issues of dengue will be interested to participate, whereby responses obtained through convenience sampling might not be representative of the overall population. However on the aspect of generalizability, there was a good demographic representation between study sample and national demographics, in terms of age, ethnicity, gender, housing type and employment status. Thus the findings may be generalized to the local population to a certain extent (Supplementary Table 9). Counter-strategies included adjusting the survey length through an initial pre-test, comparing demographics between complete and incomplete responses (Supplementary Table 9), and extensive distribution of survey. Between the completed and partially completed survey respondents, significant demographic differences observed were marital status, years staying in house and people staying in house, while the other demographic variables (such as age, ethnicity, gender, etc.) were comparable between the two groups. Incentives and survey follow-up could not be done due to the nature of the survey (one-year, part-time school project), as well as restrictions imposed by Covid-19 Safe Management Measures (SMM).

Another possible limitation is information bias, as there was no way to validate the accuracy of each input from the anonymous survey. Error from inputting wrong postal codes would result in misclassification bias. There might be recall bias as some questions involved remembering activities conducted in 2019–2020. In addition, as this is a cross-sectional study, causal associations cannot be drawn from the results. However, the associations could help re-evaluate the efficacy of current dengue campaigns amongst the local population. Another possible limitation could be that respondents were not asked if they had family or close friends who experienced dengue, which might influence knowledge or awareness of dengue. However, a similar study had revealed no association between having family member with history of dengue and an increase in respondent knowledge of dengue^57^.

### Public health implications

This study suggested that targeted public health education emphasizing on significant associated factors involving knowledge, attitudes and practices could demystify common misconceptions about dengue prevention, and enhance dengue protective effect among respondents, especially in SHS areas. Faced with potential waning dengue antibodies and located within SHS area, residents aged 41–50 years old had been identified as a high risk factor. Incentives to entice these residents to attend specially curated dengue outreach events, could enable age-specific targeted intervention aimed at identifying knowledge gaps and educating on the risk of dengue and effective mosquito prevention methods. Targeted interventions could also apply to residents with shorter duration of current residence through dengue outreach events aimed at promoting awareness of existing dengue serotype in their area and learning how to contribute to a clean and mosquito-free home environment. Building an inclusive community through the support of community leaders and volunteers will help these residents feel comfortable to engage and be pro-active towards dengue prevention. To mitigate dengue risk in local condominiums, tailored interventions mediated by both condominium staff and residents could be a foreseeable countermeasure. Lastly, further case–control and cross-sectional studies could be employed to derive the validity of K-A and A-P relationships. Significant KAP statements associated with SHS could be useful reference points to identify gaps between residents’ knowledge and their attitudes/behaviours, which in turn would allow refining of targeted public health education to bridge the potential K-A and K-P gap among SHS and NSHS residents.

## Conclusion

After a decade of integrated vector control management, and in the aftermath of the 2020 dengue epidemic, assessing current KAP among residents in Singapore has unraveled factors associated with SHS, such as residents aged 41–50, staying in condominiums, as well as residents with current residence duration of less than 10 years. Our findings would allow for fresh evaluation and refining of existing dengue public policies and strategies, and would also provide means of evidence-based assessment of current mosquito breeding (and hence, dengue) prevention strategies as well as identify any new gaps in current dengue KAPs. Significant KAP factors associated with SHS derived from our study could be further evaluated to finetune existing public education on dengue prevention in Singapore. Targeted interventions focusing on the factors associated with SHS could potentially reduce the risk of dengue in sustained hotspots in Singapore.

## Date availability

All data generated or analysed during this study are included in this published article (and its Supplementary Information files).

## Supplementary Information

Supplementary Information.
