# Supplementary material for: Knowledge, attitudes and practices of dengue prevention between dengue sustained hotspots and non-sustained hotspots in Singapore: a cross-sectional study
Source: Sci Rep. 2022 Nov 1;12:18426. doi: 10.1038/s41598-022-22776-y (PMC9626577; doi:10.1038/s41598-022-22776-y)
Supplement: Supplementary file 1 — Supplementary Information. [file 41598_2022_22776_MOESM1_ESM.docx]

# **Supplementary Material**

**Supplementary Table 1. Cronbach’s alpha for reliability testing**


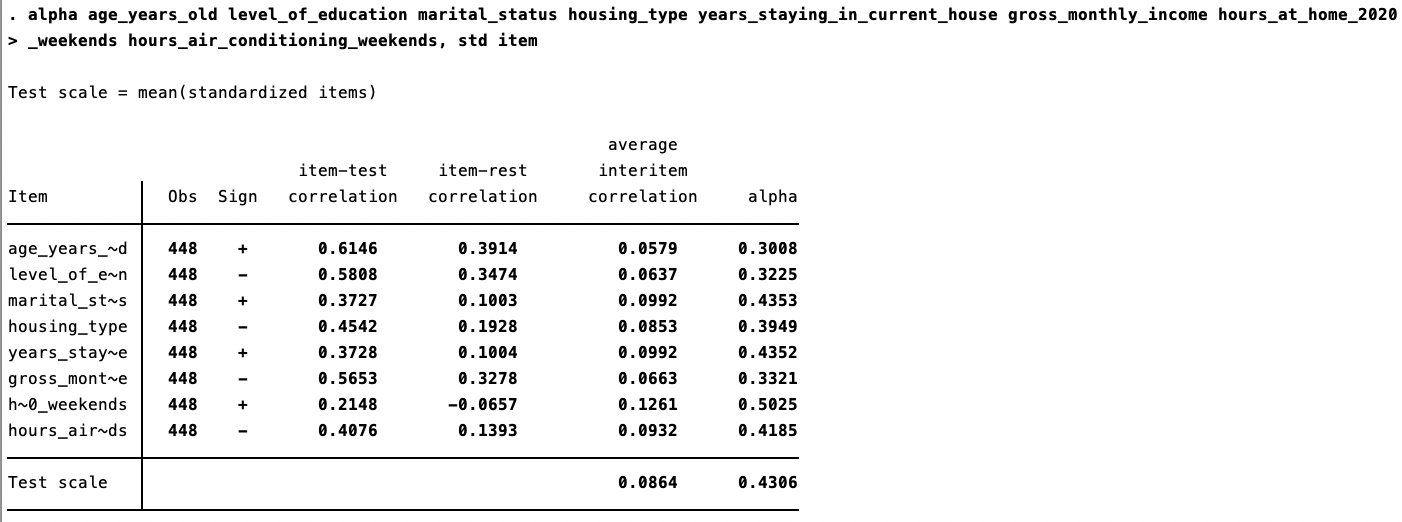


**
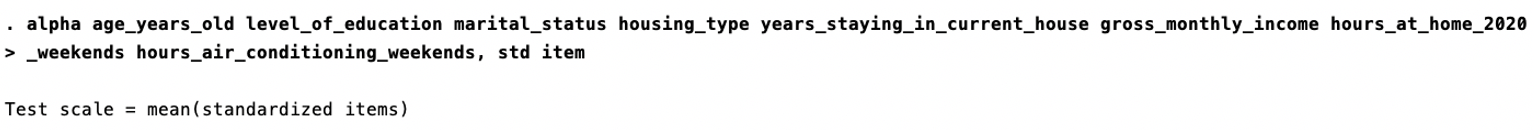
**

**Supplementary Table 2. Skewness/Kurtosis tests for Normality**

**
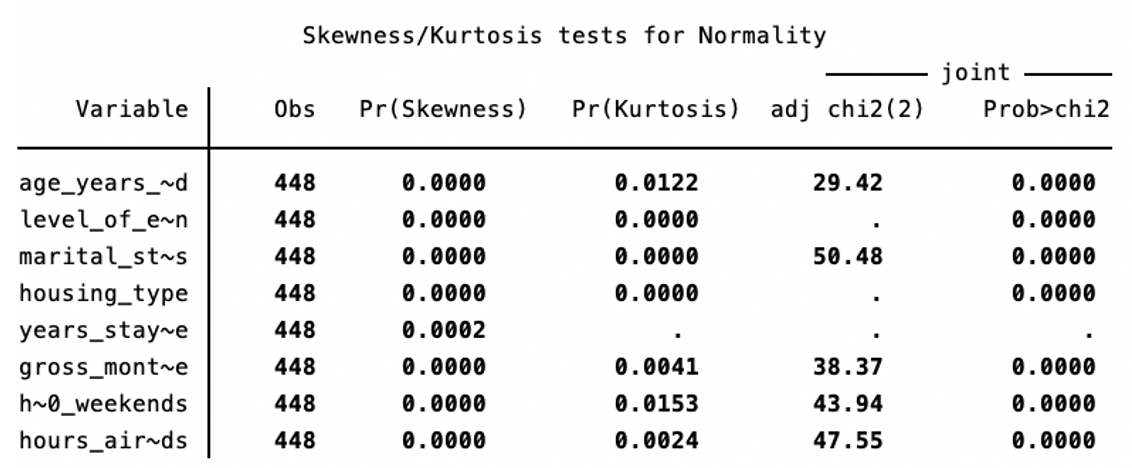
**

**
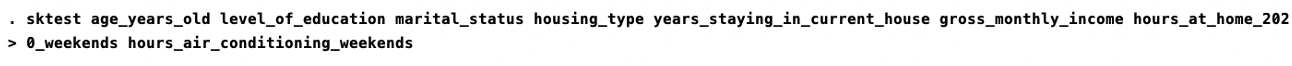
**

**Supplementary Table 3**. Demographic and characteristic differences between SHS and NSHS (full)

| **Demographic characteristic** | | **Respondents (No. %)**  All SHS NSHS | | | **χ2 /T- Test**  P-value | **Crude Odds Ratio (COR)**  OR 95% CI P-value | | | **Adjusted Odds Ratio (AOR)***  OR 95% CI P-value | | |
| --- | --- | --- | --- | --- | --- | --- | --- | --- | --- | --- | --- |
| Gender | Female | 249 (53) | 189 (55) | 60 (50) | **-** | 1.00 | - | - | 1.00 | - | - |
|  | Male | 217 (47) | 157 (45) | 60 (50) | 0.38 | 1.20 | 0.79 – 1.82 | 0.38 | 1.33 | 0.85 – 2.08 | 0.22 |
| Age | Mean^ | 41.2 | 41.0 | 41.7 | 0.60 | 1.00 | 0.98 – 1.02 | 0.87 | 1.01 | 0.95 – 1.07 | 0.86 |
|  | 31 – 40 years old |  | 121 (35) | 43 (36) | **<0.01** | 1.00 | - | - | 1.00 | - | - |
|  | 21 – 30 years old |  | 81 (23) | 17 (14) |  | 1.69 | 0.90 – 3.17 | 0.10 | 1.23 | 0.64 – 2.38 | 0.54 |
|  | 41 – 50 years old |  | 57 (17) | 36 (30) |  | 3.01 | 1.54 – 5.87 | **<0.01** | 2.50 | 1.25 – 5.03 | **0.01** |
|  | >50 years old |  | 87 (25) | 24 (20) |  | 1.31 | 0.66 – 2.62 | 0.44 | 1.78 | 0.86 – 3.70 | 0.12 |
| Ethnicity | Chinese | 324 (70) | 237 (68) | 87 (72) | 0.09 | 1.00 | - |  | 1.00 | - | - |
|  | Malay | 52 (11) | 45 (13) | 7 (6) |  | 0.42 | 0.18 – 0.98 | **0.043** | 0.41 | 0.17 – 0.98 | **0.04** |
|  | Indians and others | 90 (19) | 64 (19) | 26 (22) |  | 1.11 | 0.66 – 1.86 | 0.70 | 0.87 | 0.50 – 1.54 | 0.64 |
| Educational level | Post-Secondary | 423 (91) | 307 (89) | 116 (97) | **0.01** | 1.00 | - | - | 1.00 | - | - |
|  | Up to Secondary school | 43 (9) | 39 (11) | 4 (3) |  | 0.27 | 0.09 – 0.78 | **0.015** | 0.22 | 0.07 – 0.65 | **<0.01** |
| Marital Status | Married | 240 (51) | 159 (46) | 81 (67) | **<0.01** | 1.00 | - |  | 1.00 | - | - |
|  | Single | 213 (46) | 177 (51) | 36 (30) |  | 0.40 | 0.26 – 0.62 | **<0.01** | 0.59 | 0.33 – 1.03 | 0.06 |
|  | Divorcee/Widow(er) | 13 (3) | 10 (3) | 3 (3) |  | 0.59 | 0.16 – 2.20 | 0.43 | 1.37 | 0.31 – 5.94 | 0.68 |
| Employment Status | Working | 375 (80) | 282 (81) | 93 (77) | 0.34 | 1.00 | - |  | 1.00 | - | - |
|  | Non-working | 91 (20) | 64 (19) | 27 (23) |  | 1.28 | 0.77 – 2.12 | 0.34 | 1.31 | 0.76 – 2.28 | 0.33 |
| Housing Type | HDB & Rental Blocks | 338 (73) | 266 (77) | 72 (60) | **<0.01** | 1.00 | - |  | 1.00 | - | - |
|  | Condominium | 93 (20) | 51 (15) | 42 (35) |  | 3.04 | 1.87 – 4.94 | **<0.01** | 1.99 | 1.17 – 3.39 | **0.01** |
|  | Landed | 35 (7) | 29 (8) | 6 (5) |  | 0.76 | 0.31 –1.91 | 0.57 | 0.64 | 0.24 – 1.67 | 0.36 |
| Years staying in house | More than 10 | 237 (51) | 202 (59) | 35 (29) | **<0.01** | 1.00 | - |  | 1.00 | - | - |
|  | 5 to 10 | 83 (18) | 57 (16) | 26 (22) |  | 2.63 | 1.46 – 4.73 | **<0.01** | 2.60 | 1.42 – 4.75 | **<0.01** |
|  | 2 to 5 | 146 (31) | 87 (25) | 59 (49) |  | 3.91 | 2.40 – 6.38 | **<0.01** | 4.10 | 2.44 – 6.88 | **<0.01** |
| Gross monthly income ($) | >7,000 | 231 (49) | 158 (46) | 73 (61) | **0.01** | 1.00 | - | - | 1.00 | - | - |
|  | 3,000-6,999 | 161 (35) | 127 (37) | 34 (28) |  | 0.58 | 0.36 – 0.93 | **0.02** | 0.83 | 0.50 – 1.38 | 0.48 |
|  | <3,000 | 74 (16) | 61 (17) | 13 (11) |  | 0.46 | 0.24 – 0.89 | **0.02** | 0.76 | 0.36 – 1.57 | 0.46 |
| People staying in house | 3 - 4 people | 245 (53) | 186 (54) | 59 (49) | 0.05 | 1.00 | - | - | 1.00 | - | - |
|  | 5 or more | 128 (27) | 100 (29) | 28 (23) |  | 0.88 | 0.53 – 1.47 | 0.63 | 0.72 | 0.42 – 1.25 | 0.25 |
|  | 1 – 2 people | 93 (20) | 60 (17) | 33 (28) |  | 1.73 | 1.04 – 2.90 | **0.037** | 1.31 | 0.75 – 2.29 | 0.35 |
| Experience dengue (2019) | No | 456 (98) | 340 (98) | 116 (97) | 0.30 | 1.00 | - | - | 1.00 | - | - |
|  | Yes | 10 (2) | 6 (2) | 4 (3) |  | 1.95 | 0.54– 7.05 | 0.31 | 2.74 | 0.71 – 10.55 | 0.14 |
| Experience dengue (2020) | No | 457 (98) | 338 (98) | 119 (99) | 0.31 | 1.00 | - | - | 1.00 | - | - |
|  | Yes | 9 (2) | 8 (2) | 1 (1) |  | 0.36 | 0.04 – 2.87 | 0.33 | 0.26 | 0.03 – 2.36 | 0.23 |
| Bitten by mosquito | Yes | 317 (68) | 231 (67) | 86 (72) | 0.32 | 1.00 | - | - | 1.00 | - | - |
|  | No | 149 (32) | 115 (33) | 34 (28) |  | 0.79 | 0.50 – 1.25 | 0.32 | 0.78 | 0.48 - 1.27 | 0.32 |
| Have any disease? | No pre-existing disease | 382 (82) | 286 (83) | 96 (80) | 0.51 | 1.00 | - |  | 1.00 | - | - |
|  | Pre-existing disease | 84 (18) | 60 (17) | 24 (20) |  | 1.19 | 0.70 – 2.02 | 0.51 | 1.46 | 0.82 – 2.62 | 0.20 |
| Plant ownership | Outdoor | 227 (49) | 177 (51) | 50 (42) | 0.19 | 1.00 | - | - | 1.00 | - | - |
|  | Indoor | 102 (22) | 73 (21) | 29 (24) |  | 1.41 | 0.83 – 2.40 | 0.21 | 1.02 | 0.57 – 1.82 | 0.96 |
|  | None | 137 (29) | 196 (28) | 41 (34) |  | 1.51 | 0.93 – 2.45 | 0.09 | 1.33 | 0.79 – 2.23 | 0.29 |
| Pet Ownership | No | 349 (75) | 263 (76) | 86 (72) | 0.34 | 1.00 | - | - | 1.00 | - | - |
|  | Yes | 117 (25) | 83 (24) | 34 (28) |  | 1.25 | 0.78 – 2.00 | 0.35 | 1.25 | 0.75 - 2.06 | 0.39 |

*Adjusted for Age (category), Education Level and Years staying in home

^T-test used

**Supplementary Table 3**. Demographic and characteristic differences between SHS and NSHS (full) [cont’d]

| **Demographic characteristic** | | **Respondents (No. %)** | | | **χ2 /T- Test** | **Crude Odds Ratio (COR)** | | **Adjusted Odds Ratio (AOR)*** | | | |
| --- | --- | --- | --- | --- | --- | --- | --- | --- | --- | --- | --- |
|  | | All | SHS | NSHS | P-value | OR | 95% CI | P- value | OR | 95% CI | P-value |
| Wild animals sighted in neighbour-hood | Yes | 319 (68) | 235 (68) | 84 (70) | 0.67 | 1.00 | - | - | 1.00 | - |  |
|  | No | 147 (32) | 111 (32) | 36 (30) |  | 0.91 | 0.58 – 1.42 | 0.67 | 0.98 | 0.61 – 1.59 | 0.94 |
| Stagnant water sighted in neighbour-hood | Yes | 288 (62) | 214 (62) | 74 (62) | 0.97 | 1.00 | - | - | 1.00 | - | - |
|  | No | 178 (38) | 132 (38) | 46 (38) |  | 1.01 | 0.66 – 1.54 | 0.97 | 1.05 | 0.66 – 1.65 | 0.84 |
| Travelled in Asia | 1-2 times | 258 (55) | 199 (57) | 59 (49) | 0.26 | 1.00 | - | - | 1.00 | - | - |
|  | None | 114 (25) | 82 (24) | 32 (27) |  | 1.32 | 0.80 – 2.17 | 0.28 | 1.36 | 0.79 – 2.35 | 0.27 |
|  | More than 3 times | 94 (20) | 65 (19) | 29 (24) |  | 1.50 | 0.89 – 2.54 | 0.13 | 1.24 | 0.71 – 2.17 | 0.45 |
| Hours at home (2019, weekdays) | Mean^ | 12.3 | 12.2 | 12.6 | 0.36 | 1.02 | 0.98 – 1.07 | 0.36 | 1.01 | 0.96 – 1.06 | 0.70 |
|  | ≤12 hours | 289 (62) | 220 (64) | 69 (58) |  | 1.00 | - | - | 1.00 | - | - |
|  | >12 hours | 177 (38) | 126 (36) | 51 (42) |  | 1.29 | 0.85 – 1.97 | 0.24 | 1.16 | 0.74 – 1.83 | 0.52 |
| Hours at home (2019, weekends) | Mean^ | 15.5 | 15.3 | 16.1 | 0.14 | 1.03 | 0.99 – 1.08 | 0.14 | 1.04 | 0.99 – 1.09 | 0.11 |
|  | >12 hours | 320 (69) | 231 (67) | 89 (70) |  | 1.00 | - | - | 1.00 | - | - |
|  | ≤12 hours | 146 (31) | 115 (33) | 31 (30) |  | 0.70 | 0.44 – 1.11 | 0.13 | 0.72 | 0.43 – 1.18 | 0.19 |
| Hours at home (2020, weekdays)) | Mean^ | 15.7 | 15.5 | 16.3 | 0.14 | 1.03 | 0.99 – 1.07 | 0.14 | 1.01 | 0.97 – 1.05 | 0.69 |
|  | >12 hours | 306 (66) | 222 (64) | 84 (71) |  | 1.00 | - | - | 1.00 | - | - |
|  | ≤12 hours | 160 (34) | 124 (36) | 36 (29) |  | 0.77 | 0.49 – 1.20 | 0.25 | 0.92 | 0.57 – 1.49 | 0.74 |
| Hours at home (2020, weekends) | Mean^ | 17.5 | 17.2 | 18.3 | **0.03** | 1.05 | 1.01 – 1.10 | **0.03** | 1.04 | 0.99 – 1.10 | 0.09 |
|  | >12 hours | 382 (82) | 279 (81) | 103 (86) |  | 1.00 | - | - | 1.00 | - | - |
|  | ≤12 hours | 84 (18) | 67 (19) | 17 (14) |  | 0.69 | 0.39 – 1.23 | 0.20 | 0.75 | 0.41 – 1.39 | 0.37 |
| Hours air conditioning (weekdays) | Mean^ | 6.1 | 5.9 | 6.6 | 0.23 | 1.02 | 0.99 – 1.06 | 0.23 | 0.99 | 0.95 – 1.04 | 0.80 |
|  | ≤12 hours | 430 (92) | 321 (93) | 109 (91) |  | 1.00 | - | - | 1.00 | - | - |
|  | >12 hours | 36 (8) | 25 (7) | 11 (9) |  | 1.30 | 0.62 – 2.72 | 0.49 | 0.86 | 0.39 – 1.89 | 0.71 |
| Hours air conditioning (weekends) | Mean^ | 6.2 | 5.9 | 7.2 | **0.03** | 1.04 | 1.00 – 1.08 | **0.03** | 1.01 | 0.97 – 1.06 | 0.48 |
|  | ≤12 hours | 419 (90) | 317 (92) | 102 (85) |  | 1.00 | - | **-** | 1.00 | - | - |
|  | >12 hours | 47 (10) | 29 (8) | 18 (15) |  | 1.93 | 1.03 – 3.62 | **0.04** | 1.36 | 0.70 – 2.67 | 0.36 |

*Adjusted for Age (category), Education Level and Years staying in home

^T-test used

**Supplementary Table 4**. Comparison of Knowledge topics between SHS and NSHS respondents (full)

| **Knowledge characteristic^** | **Correct response, No. (%)** | | | **χ2 Test** | **Crude Odds Ratio (COR)** | | | **Adjusted Odds Ratio (AOR)*** | | |
| --- | --- | --- | --- | --- | --- | --- | --- | --- | --- | --- |
|  | Total | SHS | NSHS | P-value | OR | 95% CI | P-value | OR | 95% CI | P-value |
| **Mosquitoes** |  |  |  |  |  |  |  |  |  |  |
| All mosquitoes transmit dengue virus | 399 (86) | 298 (86) | 101 (84) | 0.60 | 0.86 | 0.48 – 1.52 | 0.60 | 0.59 | 0.31 – 1.11 | 0.10 |
| Main vectors for dengue are *Aedes* aegypti and *Aedes* albopictus mosquitoes | 397 (85) | 296 (86) | 101 (84) | 0.71 | 0.90 | 0.51 – 1.59 | 0.71 | 0.81 | 0.44 – 1.50 | 0.50 |
| Mosquitoes can breed indoors and outdoors | 450 (97) | 334 (97) | 116 (97) | 0.94 | 1.04 | 0.33 – 3.29 | 0.94 | 0.65 | 0.19 – 2.25 | 0.50 |
| Mosquitoes likes to breed in clean water | 192 (41) | 146 (42) | 46 (38) | 0.46 | 0.85 | 0.56 – 1.30 | 0.46 | 0.91 | 0.57 – 1.43 | 0.67 |
| Eggs of mosquitoes can survive up to 6 months in dry conditions | 252 (54) | 197 (57) | 55 (46) | **0.04** | 0.64 | 0.42 – 0.97 | **0.04** | 0.64 | 0.41 – 0.99 | **0.046** |
| Life cycle of a mosquito is one week | 279 (60) | 210 (61) | 69 (58) | 0.54 | 0.88 | 0.57 – 1.34 | 0.54 | 0.88 | 0.56 – 1.38 | 0.57 |
| Distance of flight of *Aedes* mosquitoes is 150m | 210 (45) | 166 (48) | 44 (37) | **0.03** | 0.63 | 0.41 – 0.96 | **0.03** | 0.62 | 0.39 – 0.98 | **0.041** |
| Female mosquitoes bite on human | 409 (88) | 305 (88) | 104 (87) | 0.67 | 0.87 | 0.47 – 1.62 | 0.67 | 0.91 | 0.47 – 1.75 | 0.77 |
| Female mosquitoes bite on animals | 318 (68) | 236 (68) | 82 (68) | 0.98 | 1.01 | 0.64 – 1.57 | 0.98 | 0.90 | 0.56 – 1.45 | 0.68 |
| Stagnant water is the main source of mosquito breeding | 438 (94) | 326 (94) | 112 (93) | 0.73 | 0.86 | 0.37 – 2.00 | 0.73 | 0.68 | 0.27 – 1.69 | 0.41 |
| **Dengue** |  |  |  |  |  |  |  |  |  |  |
| If my family member has dengue before, my chances of getting dengue is much higher | 196 (42) | 150 (43) | 46 (38) | 0.34 | 0.81 | 0.53 – 1.24 | 0.34 | 0.77 | 0.49 – 1.21 | 0.26 |
| Dengue only occurs during the rainy season | 401 (86) | 298 (86) | 103 (86) | 0.94 | 0.98 | 0.54 – 1.77 | 0.94 | 0.87 | 0.46 – 1.66 | 0.68 |
| You can only be infected with dengue once | 424 (91) | 317 (92) | 107 (89) | 0.42 | 0.75 | 0.38 – 1.50 | 0.42 | 0.70 | 0.33 – 1.46 | 0.34 |
| Mosquitoes transmitting dengue only bites during daytime | 315 (68) | 228 (66) | 87 (73) | 0.18 | 1.36 | 0.86 – 2.16 | 0.18 | 1.36 | 0.83 – 2.23 | 0.22 |
| **Mosquito Breeding Area** |  |  |  |  |  |  |  |  |  |  |
| Potted plants with hardened soil | 291 (62) | 222 (64) | 69 (58) | 0.19 | 0.76 | 0.49 – 1.15 | 0.20 | 0.76 | 0.48 – 1.20 | 0.24 |
| Gulley traps | 396 (85) | 296 (86) | 100 (83) | 0.56 | 0.84 | 0.48 – 1.49 | 0.56 | 0.74 | 0.40 – 1.37 | 0.34 |
| Open container with stagnant water | 454 (97) | 339 (98) | 115 (96) | 0.20 | 0.47 | 0.15 – 1.53 | 0.21 | 0.25 | 0.06 – 0.98 | **0.046** |
| **Signs and Symptoms** |  |  |  |  |  |  |  |  |  |  |
| Nose bleeding, bloody diarrhea, vomiting blood | 206 (44) | 152 (44) | 54 (45) | 0.84 | 1.04 | 0.69 – 1.59 | 0.84 | 1.05 | 0.67 – 1.65 | 0.82 |
| Fever for more than 2 days | 437 (94) | 321 (93) | 116 (97) | 0.13 | 2.26 | 0.77 – 6.63 | 0.14 | 2.12 | 0.68 – 6.54 | 0.19 |
| Headache, pain behind the eyes | 414 (89) | 310 (90) | 104 (87) | 0.38 | 0.75 | 0.40 – 1.42 | 0.38 | 0.75 | 0.38 – 1.48 | 0.41 |
| Joint pains | 435 (93) | 323 (93) | 112 (93) | 0.99 | 1.00 | 0.43 – 2.29 | 0.99 | 1.02 | 0.42 – 2.46 | 0.97 |
| Skin rash | 371 (80) | 271 (78) | 100 (83) | 0.24 | 1.38 | 0.80 – 2.38 | 0.24 | 1.31 | 0.73 – 2.33 | 0.36 |
| Asymptomatic (no signs and symptoms) | 161 (35) | 125 (36) | 36 (30) | 0.22 | 0.76 | 0.48 – 1.19 | 0.23 | 0.79 | 0.49 – 1.28 | 0.35 |
| **Prevent Dengue** |  |  |  |  |  |  |  |  |  |  |
| Applying mosquito repellent spray / mosquito repellent cream | 429 (92) | 320 (92) | 109 (91) | 0.56 | 0.81 | 0.39 – 1.68 | 0.57 | 0.75 | 0.34 – 1.65 | 0.48 |
| Using mosquito nets | 434 (93) | 327 (95) | 107 (89) | 0.046 | 0.48 | 0.23 – 1.00 | 0.05 | 0.57 | 0.25 – 1.29 | 0.18 |
| Using mosquito repellent coil | 410 (88) | 312 (90) | 98 (82) | 0.01 | 0.49 | 0.27 – 0.87 | 0.02 | 0.56 | 0.30 – 1.04 | 0.07 |
| Removing water in flowerpot/plates | 456 (98) | 339 (98) | 117 (98) | 0.76 | 0.81 | 0.20 – 3.17 | 0.76 | 0.72 | 0.17 – 3.06 | 0.65 |
| Not growing any plants - | 199 (43) | 151 (44) | 48 (40) | 0.49 | 0.86 | 0.56 – 1.31 | 0.49 | 0.81 | 0.52 – 1.28 | 0.38 |
| Removing / changing water containers daily | 446 (96) | 332 (96) | 114 (95) | 0.66 | 0.80 | 0.30 – 2.13 | 0.66 | 0.82 | 0.29 – 2.36 | 0.72 |
| **Treat Dengue** |  |  |  |  |  |  |  |  |  |  |
| Antibiotics - | 238 (51) | 170 (49) | 68 (57) | 0.16 | 1.35 | 0.89 – 2.06 | 0.16 | 1.06 | 0.68 – 1.66 | 0.80 |
| Paracetamol (Panadol) | 289 (62) | 211 (61) | 78 (65) | 0.44 | 1.19 | 0.77 – 1.83 | 0.44 | 1.04 | 0.66 – 1.65 | 0.87 |
| Intravenous fluid rehydration | 346 (74) | 252 (73) | 94 (78) | 0.24 | 1.35 | 0.82 – 2.21 | 0.24 | 1.18 | 0.70 – 2.01 | 0.54 |
| Vaccination | 115 (25) | 80 (23) | 35 (29) | 0.19 | 1.37 | 0.86 – 2.18 | 0.19 | 1.39 | 0.85 – 2.29 | 0.19 |

*Adjusted for Age (category), Education Level and Years staying in home

^Correct response gives 1 point; Wrong or Neutral response give 0 points

**Supplementary Table 5.** Mean Scores of KAP sub-sections between SHS and NHS areas

| **Characteristic Section/Sub-section**  **(no. of questions)** | **Mean Score** | | | **T-Test** | **Crude Odds Ratio (COR)** | | | **Adjusted Odds Ratio (AOR)*** | | |
| --- | --- | --- | --- | --- | --- | --- | --- | --- | --- | --- |
|  | All | SHS | NSHS | P-value | OR | 95% CI | P-value | OR | 95% CI | P-value |
| **Knowledge^ (33)** | 24.05 | 24.66 | 24.37 | 0.41 | 0.97 | 0.91 – 1.04 | 0.41 | 0.95 | 0.89 – 1.02 | 0.18 |
| Mosquitoes (10) | 7.18 | 7.27 | 6.92 | 0.08 | 0.91 | 0.82 – 1.01 | 0.08 | 0.89 | 0.79 – 0.99 | **0.04** |
| Dengue (4) | 2.87 | 2.87 | 2.86 | 0.91 | 0.99 | 0.91 – 1.21 | 0.91 | 0.96 | 0.76 – 1.19 | 0.69 |
| Mosquito Breeding Area (3) | 2.45 | 2.48 | 2.37 | 0.17 | 0.83 | 0.63 – 1.08 | 0.17 | 0.78 | 0.58 – 1.05 | 0.10 |
| Signs and Symptoms (6) | 4.34 | 4.34 | 4.35 | 0.95 | 1.00 | 0.86 – 1.17 | 0.95 | 1.01 | 0.85 – 1.19 | 0.95 |
| Prevent Dengue (6) | 5.09 | 5.15 | 4.94 | 0.06 | 0.84 | 0.69 – 1.01 | 0.06 | 0.84 | 0.69 – 1.03 | 0.10 |
| Treat Dengue (4) | 2.12 | 2.06 | 2.29 | 0.06 | 1.20 | 0.99 – 1.44 | 0.06 | 1.10 | 0.90 – 1.34 | 0.36 |
| **Attitudes# (14)** | 10.26 | 10.38 | 10.16 | 0.28 | 0.94 | 0.84 – 1.05 | 0.28 | 0.90 | 0.79 – 1.01 | 0.08 |
| Barrier (4) | 2.55 | 2.58 | 2.45 | 0.17 | 0.85 | 0.67 – 1.07 | 0.17 | 0.73 | 0.57 – 0.95 | **0.02** |
| Efficacy (6) | 4.54 | 4.53 | 4.58 | 0.62 | 1.05 | 0.86 – 1.29 | 0.62 | 1.01 | 0.81 – 1.26 | 0.95 |
| Risk (4) | 3.18 | 3.17 | 3.19 | 0.84 | 1.02 | 0.83 – 1.26 | 0.84 | 0.98 | 0.78 – 1.22 | 0.83 |
| **Practices+ (17)** | 9.15 | 9.27 | 8.80 | 0.22 | 0.96 | 0.91 – 1.02 | 0.22 | 0.95 | 0.89 – 1.02 | 0.16 |
| Frequency (9) | 4.88 | 4.90 | 4.80 | 0.65 | 0.98 | 0.89 – 1.08 | 0.65 | 0.97 | 0.88 – 1.08 | 0.62 |
| Adherence (8) | 4.27 | 4.36 | 4.00 | 0.10 | 0.92 | 0.83 – 1.02 | 0.10 | 0.90 | 0.80 – 1.01 | 0.07 |

*Adjusted for Age (category), Education Level and Years staying in home

^Correct response gives 1 point; Wrong or Neutral response give 0 points

#Positive response gives 1 point; Negative or Neutral response give 0 points

+Positive behaviour gives 1 point; Negative behaviour gives 0 points; Correct score gives 1 point; Incorrect or Neutral score give 0 points

**Supplementary Table 6**. Comparison of Attitude topics between SHS and NSHS respondents (full)

| **Attitudes characteristic^** | **Positive response No. (%)** | | | **χ2 Test** | **Crude Odds Ratio (COR)** | | | **Adjusted Odds Ratio (AOR)*** | | |
| --- | --- | --- | --- | --- | --- | --- | --- | --- | --- | --- |
|  | All | SHS | NSHS | P-value | OR | 95% CI | P-value | OR | 95% CI | P-value |
| **How do you feel about the following statements?** |  |  |  |  |  |  |  |  |  |  |
| Dengue is a serious illness that I am concerned with | 422 (91) | 311 (90) | 111 (93) | 0.40 | 1.39 | 0.65 – 2.98 | 0.40 | 1.16 | 0.52 – 2.60 | 0.71 |
| I am at risk of dengue fever | 285 (61) | 214 (62) | 71 (59) | 0.60 | 0.89 | 0.59 – 1.37 | 0.60 | 0.81 | 0.52 – 1.28 | 0.37 |
| I am afraid of getting dengue | 337 (72) | 248 (72) | 89 (74) | 0.60 | 1.13 | 0.71 – 1.82 | 0.60 | 1.15 | 0.69 – 1.90 | 0.59 |
| I am responsible to keep my place free of mosquito breeding sites | 446 (96) | 334 (97) | 112 (93) | 0.14 | 0.50 | 0.20 – 1.26 | 0.14 | 0.37 | 0.13 – 0.99 | **0.049** |
| I regularly check dengue situation or hotspot using National Environment Agency (NEA)'s website | 227 (49) | 167 (48) | 60 (50) | 0.74 | 1.07 | 0.71 – 1.62 | 0.74 | 0.99 | 0.63 – 1.57 | 0.99 |
| Chemical fogging is enough to prevent dengue | 319 (68) | 240 (69) | 79 (66) | 0.47 | 0.85 | 0.55 – 1.32 | 0.47 | 0.73 | 0.45 – 1.18 | 0.20 |
| Reducing *Aedes* mosquitoes is the only way to prevent dengue | 216 (46) | 173 (50) | 43 (36) | **<0.01** | 0.56 | 0.36 – 0.86 | **<0.01** | 0.53 | 0.33 – 0.84 | **<0.01** |
| If I have dengue symptoms, I will seek immediate treatment | 430 (92) | 325 (94) | 105 (88) | **0.02** | 0.45 | 0.23 – 0.91 | **0.03** | 0.39 | 0.18 – 0.84 | **0.02** |
| It is necessary to be hospitalized if suffering from dengue fever | 175 (38) | 134 (39) | 41 (34) | 0.37 | 0.82 | 0.53 – 1.27 | 0.37 | 0.71 | 0.45 – 1.13 | 0.15 |
| I will not visit a dengue patient in the hospital | 354 (76) | 266 (77) | 88 (73) | 0.43 | 0.83 | 0.51 – 1.33 | 0.43 | 0.66 | 0.39 – 1.11 | 0.12 |
| Healthy people will never get dengue | 436 (94) | 324 (94) | 112 (93) | 0.91 | 0.95 | 0.41 – 2.20 | 0.91 | 0.80 | 0.32 – 1.96 | 0.62 |
| Removing *Aedes* mosquitoes breeding sites prevent dengue fever among my family | 395 (85) | 290 (84) | 105 (88) | 0.33 | 1.35 | 0.73 – 2.49 | 0.33 | 1.29 | 0.68 – 2.47 | 0.44 |
| I have a role to play in preventing dengue | 450 (97) | 336 (97) | 114 (95) | 0.27 | 0.57 | 0.20 – 1.59 | 0.28 | 0.48 | 0.16 – 1.48 | 0.20 |
| I will take the dengue vaccine as recommended by Ministry of Health | 257 (55) | 194 (56) | 63 (53) | 0.50 | 0.87 | 0.57 – 1.31 | 0.50 | 0.86 | 0.55 – 1.35 | 0.52 |

*Adjusted for Age, Education Level and Years staying in home

^Positive response gives 1 point; Negative or Neutral response give 0 points

**Supplementary Table 7**. Comparison of Practice topics between SHS and NSHS respondents (full)

| **Practices characteristic** | **Positive behaviour /Correct Response**  **No. (%)** | | | **χ2 Test** | **Crude Odds Ratio (COR)** | | | **Adjusted Odds Ratio (AOR)*** | | |
| --- | --- | --- | --- | --- | --- | --- | --- | --- | --- | --- |
|  | All | SHS | NSHS | P-value | OR | 95% CI | P-value | OR | 95% CI | P-value |
| **Frequency#** |  |  |  |  |  |  |  |  |  |  |
| Changing water in water storing containers (e.g. plastic bottles or flowerpots/plates) | 250 (54) | 190 (55) | 60 (50) | 0.35 | 0.82 | 0.54 – 1.24 | 0.35 | 0.89 | 0.57– 1.39 | 0.62 |
| Covering tightly water storing containers (e.g. plastic bottles or flowerpots/plates) when not in use | 317 (68) | 236 (68) | 81 (68) | 0.89 | 0.97 | 0.62 – 1.51 | 0.89 | 0.97 | 0.60 – 1.56 | 0.90 |
| Applying abate in gulley traps or water storing containers | 114 (24) | 87 (25) | 27 (23) | 0.56 | 0.86 | 0.53 – 1.41 | 0.56 | 0.91 | 0.54 – 1.55 | 0.74 |
| Rear fish | 361 (77) | 270 (78) | 91 (76) | 0.62 | 0.88 | 0.54 – 1.44 | 0.62 | 0.74 | 0.43 – 1.27 | 0.28 |
| Overturning or removing flowerpot plates | 241 (52) | 183 (53) | 58 (48) | 0.39 | 0.83 | 0.55 – 1.26 | 0.39 | 0.89 | 0.57 – 1.40 | 0.62 |
| Using mosquito repellent coils or other mosquito repellent devices in home (eg. Electric repellent) | 114 (24) | 86 (25) | 28 (23) | 0.74 | 0.92 | 0.56 – 1.50 | 0.74 | 0.92 | 0.54 – 1.57 | 0.77 |
| Applying insect repellent (eg. Spray, patch, repellent) on body when stepping outside of house | 115 (25) | 88 (25) | 27 (23) | 0.52 | 0.85 | 0.52 – 1.39 | 0.52 | 0.83 | 0.49 – 1.41 | 0.49 |
| Keeping inside of house clean and tidy by removing all rubbish daily | 397 (85) | 289 (84) | 108 (90) | 0.09 | 1.78 | 0.92 – 3.44 | 0.09 | 1.55 | 0.78 – 3.09 | 0.21 |
| Keeping environment outside house clean by ensuring surrounding of house is free from rubbish | 364 (78) | 268 (77) | 96 (80) | 0.56 | 1.16 | 0.70 – 1.95 | 0.56 | 1.05 | 0.61 – 1.81 | 0.85 |
| **Agreement^** |  |  |  |  |  |  |  |  |  |  |
| Go to clinic / hospital immediately | 404 (87) | 302 (87) | 102 (85) | 0.53 | 0.83 | 0.46 – 1.49 | 0.53 | 0.80 | 0.43 – 1.51 | 0.50 |
| Go to clinic / hospital only if symptoms worsen | 250 (54) | 194 (56) | 56 (47) | 0.08 | 0.69 | 0.45 – 1.04 | 0.08 | 0.61 | 0.39 – 0.95 | **0.03** |
| Self-medicate | 118 (25) | 89 (26) | 29 (24) | 0.74 | 0.92 | 0.57 – 1.49 | 0.74 | 0.82 | 0.49 – 1.37 | 0.45 |
| Communicating with Dengue volunteers or NEA officers to learn more about dengue | 180 (39) | 146 (42) | 34 (28) | **<0.01** | 0.54 | 0.35 – 0.85 | **<0.01** | 0.53 | 0.32 – 0.88 | **0.01** |
| Sharing with neighbours on mosquito preventive measures and how to fight dengue | 165 (35) | 131 (38) | 34 (28) | 0.06 | 0.65 | 0.41 – 1.02 | 0.06 | 0.65 | 0.39 – 1.09 | 0.10 |
| Sharing with family members on mosquito preventive measures and how to fight dengue | 312 (67) | 231 (67) | 81 (68) | 0.88 | 1.03 | 0.66 – 1.61 | 0.88 | 0.99 | 0.61 – 1.61 | 0.98 |
| Informing Town Council on maintenance issues in common areas that might lead to mosquito breeding | 287 (62) | 211 (61) | 76 (63) | 0.65 | 1.11 | 0.72 – 1.70 | 0.65 | 1.09 | 0.68 – 1.75 | 0.71 |
| Informing NEA on mosquito-related feedback or queries | 274 (59) | 206 (60) | 68 (57) | 0.58 | 0.89 | 0.58 – 1.35 | 0.58 | 0.85 | 0.53 – 1.36 | 0.50 |

*Adjusted for Age, Education Level and Years staying in home

^Correct score gives 1 point ; Incorrect or Neutral score give 0 points

#Positive behaviour gives 1 point ; Negative behaviour gives 0 points

**Supplementary Table 8**. Significant factors associated with SHS

| **Category** | **Sub-Category** | **Significant SHS Associated Factors** | **χ2 Test** | **Crude Odds Ratio (COR)** | | | **Adjusted Odds Ratio (AOR)*** | | |
| --- | --- | --- | --- | --- | --- | --- | --- | --- | --- |
|  |  |  | P-value | OR | 95% CI | P-value | OR | 95% CI | P-value |
| **Demographics** |  |  |  |  |  |  |  |  |  |
|  | Age | 41-50 years old | **<0.01** | 3.01 | 1.54 – 5.87 | **<0.01** | 2.50 | 1.25 – 5.03 | **0.01** |
|  | Ethnicity | Malay | 0.09 | 0.42 | 0.18 – 0.98 | **0.043** | 0.41 | 0.17 – 0.98 | **0.04** |
|  | Housing Type | Condominium | **<0.01** | 3.04 | 1.87 – 4.94 | **<0.01** | 1.99 | 1.17 – 3.39 | **0.01** |
|  | Years staying in house | 5 - 10 | **<0.01** | 2.63 | 1.46 – 4.73 | **<0.01** | 2.60 | 1.42 – 4.75 | **<0.01** |
|  | Years staying in house | 2 – 5 | **<0.01** | 3.91 | 2.40 – 6.38 | **<0.01** | 4.10 | 2.44 – 6.88 | **<0.01** |
| **Knowledge** |  |  |  |  |  |  |  |  |  |
|  | Mosquitoes | Eggs of mosquitoes can survive up to 6 months in dry conditions | **0.04** | 0.64 | 0.42 – 0.97 | **0.04** | 0.64 | 0.41 – 0.99 | **0.046** |
|  | Mosquitoes | Distance of flight of *Aedes* mosquitoes is 150 metres | **0.03** | 0.63 | 0.41 – 0.96 | **0.03** | 0.62 | 0.39 – 0.98 | **0.041** |
|  | Mosquito Breeding Areas | Mosquito can breed in open container with stagnant water | 0.20 | 0.47 | 0.15 – 1.53 | 0.21 | 0.25 | 0.06 – 0.98 | **0.046** |
| **Attitudes** |  |  |  |  |  |  |  |  |  |
|  | Efficacy | I am responsible to keep my place free from mosquito breeding sites | 0.14 | 0.50 | 0.20 – 1.26 | 0.14 | 0.37 | 0.13 – 0.99 | **0.049** |
|  | Efficacy | Reducing *Aedes* mosquitoes is the only way to prevent dengue | **<0.01** | 0.56 | 0.36 – 0.86 | **<0.01** | 0.53 | 0.33 – 0.84 | **<0.01** |
|  | Barrier | If I have dengue symptoms, I will seek immediate treatment | **0.02** | 0.45 | 0.23 – 0.91 | **0.03** | 0.39 | 0.18 – 0.84 | **0.02** |
| **Practices** |  |  |  |  |  |  |  |  |  |
|  | Adherence | Go to clinic / hospital even without severe symptoms | 0.08 | 0.69 | 0.45 – 1.04 | 0.08 | 0.61 | 0.39 – 0.95 | **0.03** |
|  | Adherence | Communicating with Dengue volunteers or NEA officers to learn more about dengue | **<0.01** | 0.54 | 0.35 – 0.85 | **<0.01** | 0.53 | 0.32 – 0.88 | **0.01** |

*Adjusted for Age, Education Level and Years staying in home

**Supplementary Table 9.** Comparison of complete, incomplete respondents and national demographics

*National Demographics data referenced from latest available years (2019 – 2020) in data.gov.sg and singstat.gov.sg

| **Demographic characteristic** | | **Complete (n = 466)** | | **Incomplete (n = 49)** | | **National Demographics*** | **Chi square/T-test** |
| --- | --- | --- | --- | --- | --- | --- | --- |
|  | | No. | % | No. | % | % | P-value |
| Gender | Male | 218 | 47 | 24 | 49 | 48 | 0.77 |
|  | Female | 248 | 53 | 25 | 51 | 52 |  |
| Age | Mean | 41.2 | - | 41.7 | - | 42.2 | 0.78 |
| Ethnicity | Chinese | 323 | 70 | 36 | 74 | 77 | 0.53 |
|  | Malay | 53 | 11 | 3 | 6 | 12 |  |
|  | Indians and others | 90 | 19 | 10 | 20 | 11 |  |
| Educational level | Up to Secondary school | 43 | 9 | 7 | 14 | 58 | 0.26 |
|  | Post-Secondary | 423 | 91 | 42 | 86 | 42 |  |
| Marital Status | Single | 213 | 45 | 11 | 22 | 31 | **<0.01** |
|  | Married | 240 | 52 | 36 | 74 | 59 |  |
|  | Divorcee/Widow/Widower | 13 | 3 | 2 | 4 | 10 |  |
| Employment Status | Working | 374 | 80 | 34 | 69 | 68 | 0.07 |
|  | Non-working | 92 | 20 | 15 | 31 | 32 |  |
| Housing Type | HDB & Rental Blocks | 339 | 73 | 31 | 63 | 79 | 0.22 |
|  | Condominium | 93 | 20 | 15 | 31 | 16 |  |
|  | Landed | 34 | 7 | 3 | 6 | 5 |  |
| Years staying in house | Less than 5 | 146 | 31 | 24 | 49 | - | **0.02** |
|  | 5 to 10 | 83 | 18 | 10 | 20 | - |  |
|  | More than 10 | 237 | 51 | 15 | 31 | - |  |
| Gross monthly income ($) | <3,000 | 74 | 16 | 10 | 20 | - | 0.34 |
|  | 3,000-6,999 | 161 | 35 | 12 | 25 | - |  |
|  | >7,000 | 231 | 49 | 27 | 55 | - |  |
| People staying in house | 1 – 2 people | 93 | 20 | 17 | 35 | 39 | **0.046** |
|  | 3 - 4 people | 245 | 53 | 19 | 39 | 41 |  |
|  | 5 or more | 128 | 27 | 13 | 26 | 20 |  |

**
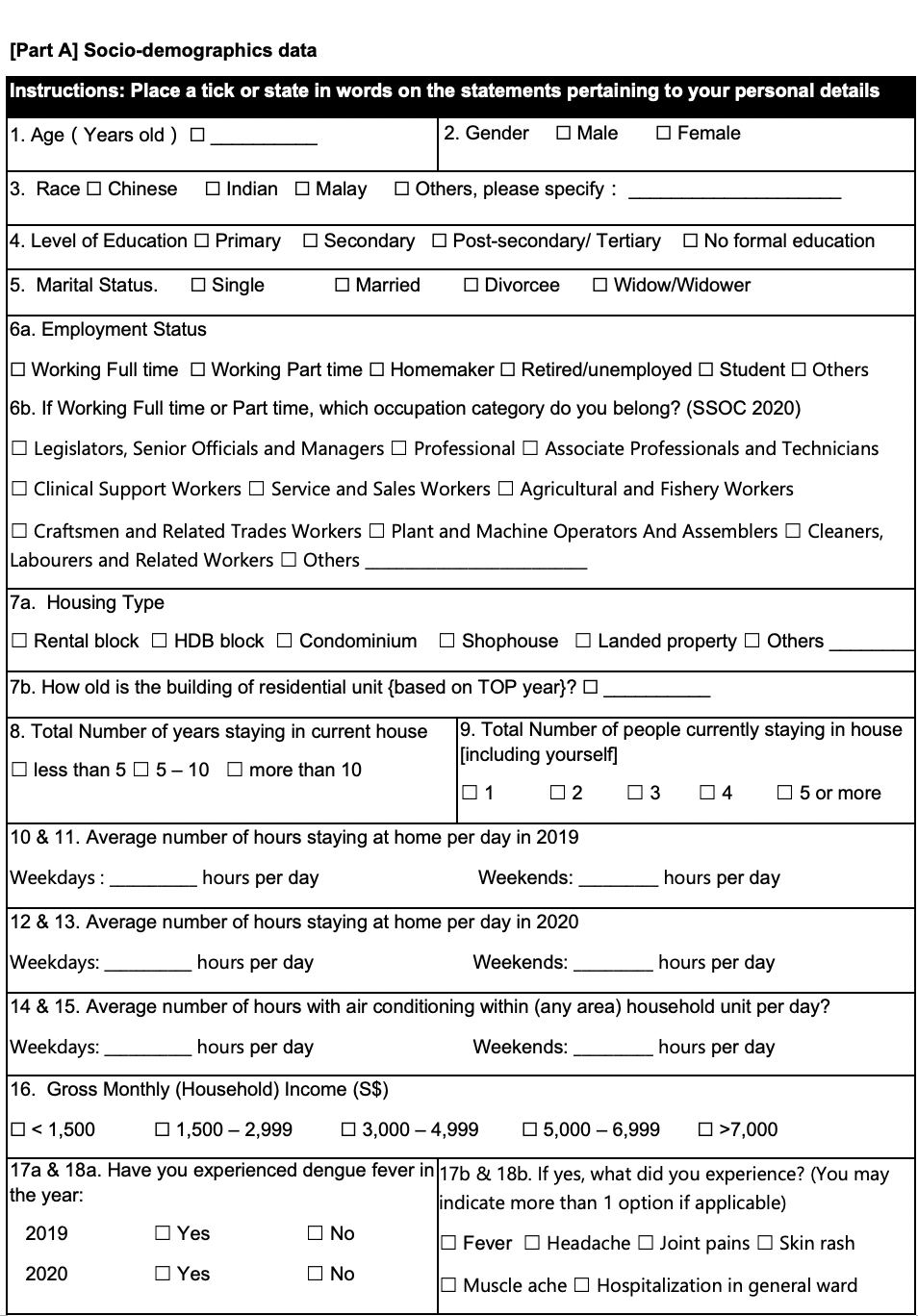

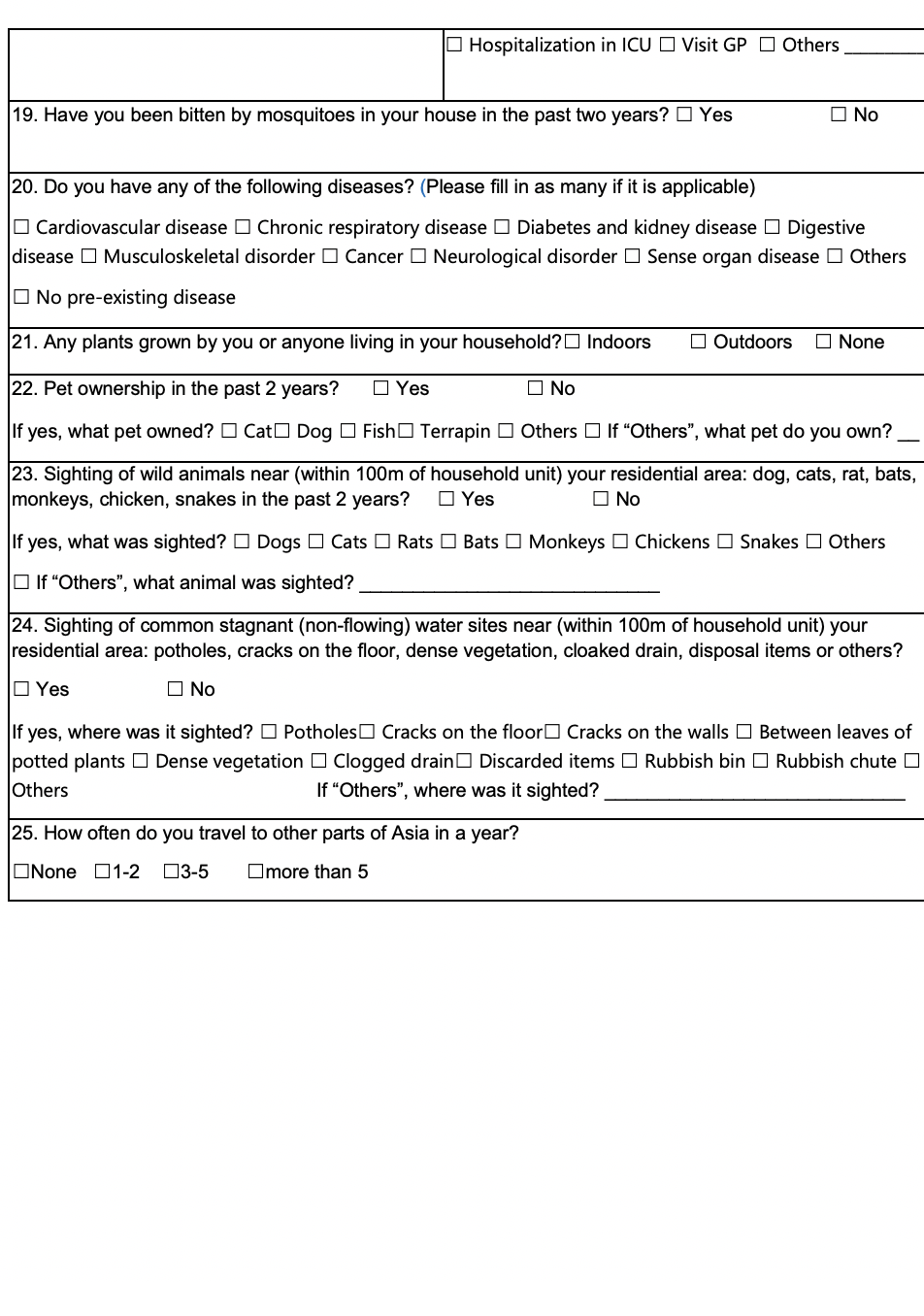
**

**
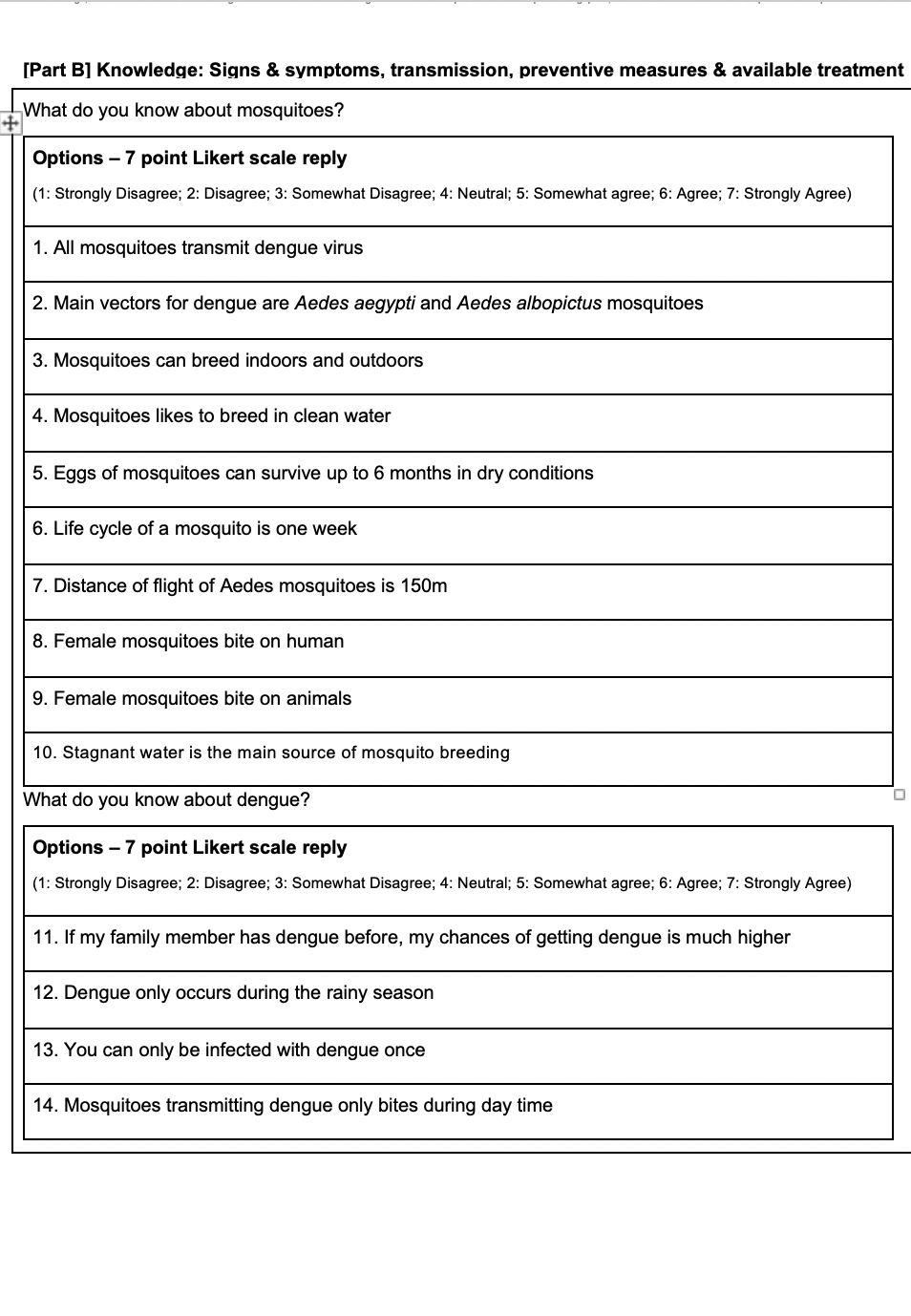

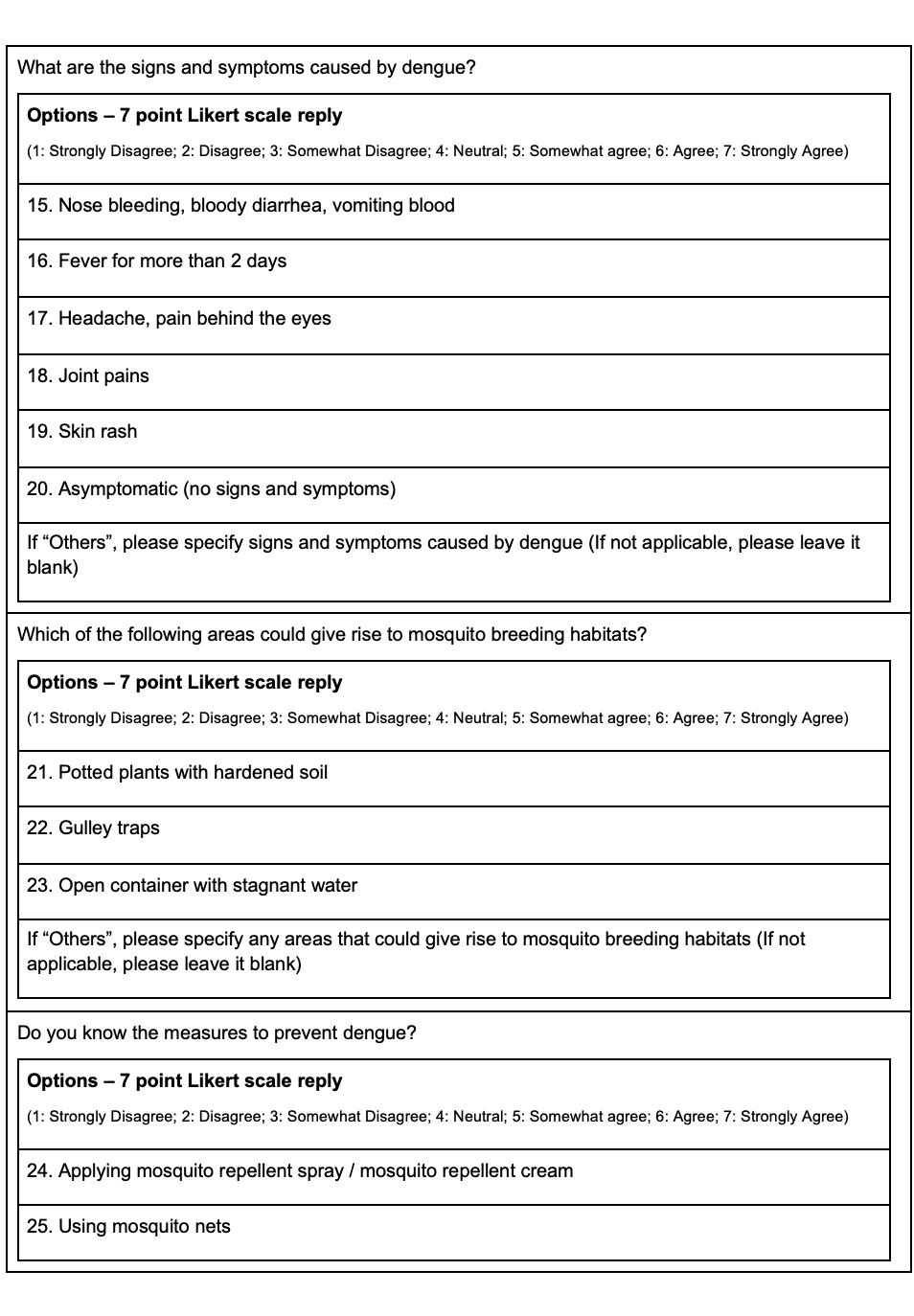
**

**Supplementary Figure 1.** KAP Questionnaire (English)

**
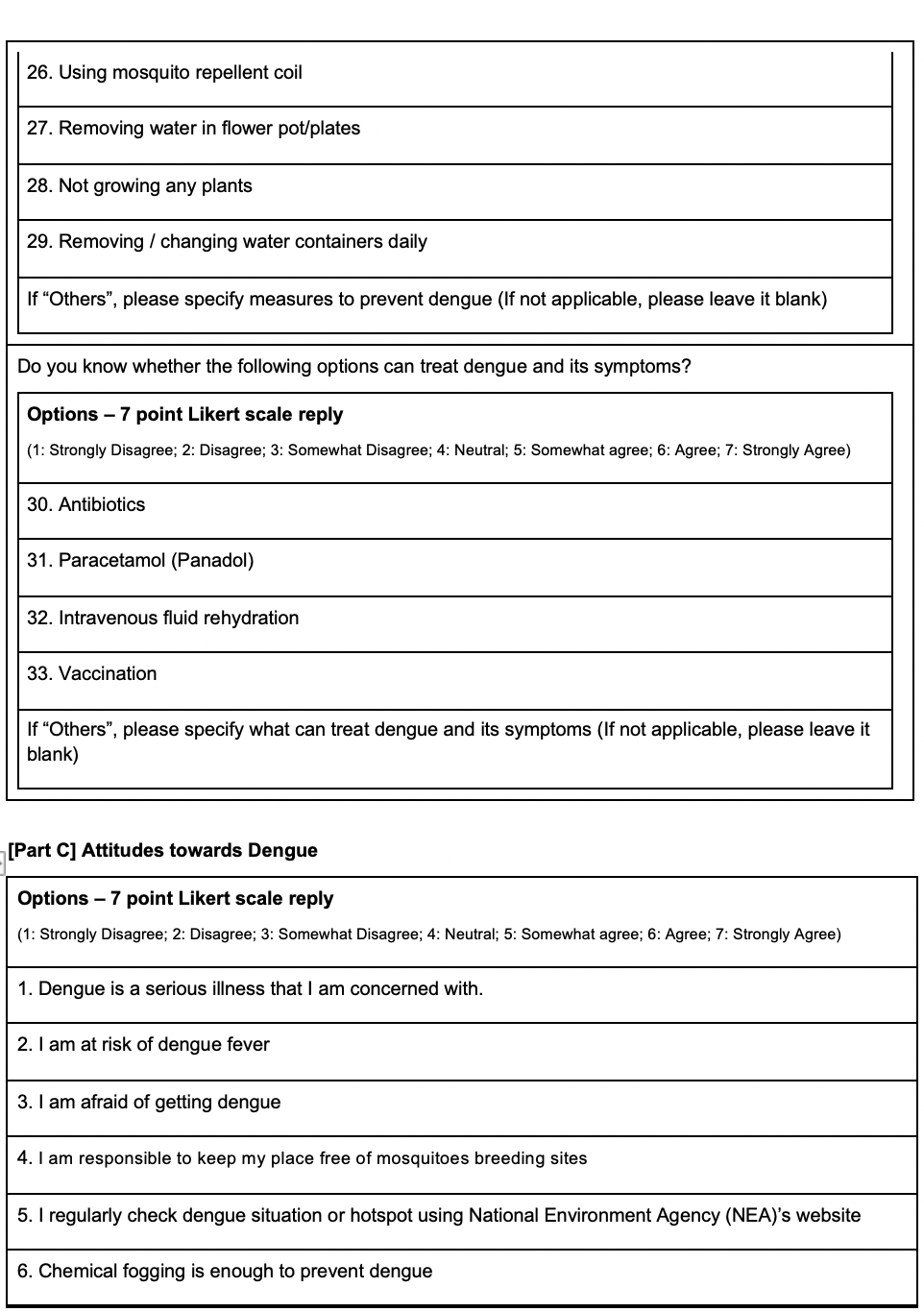

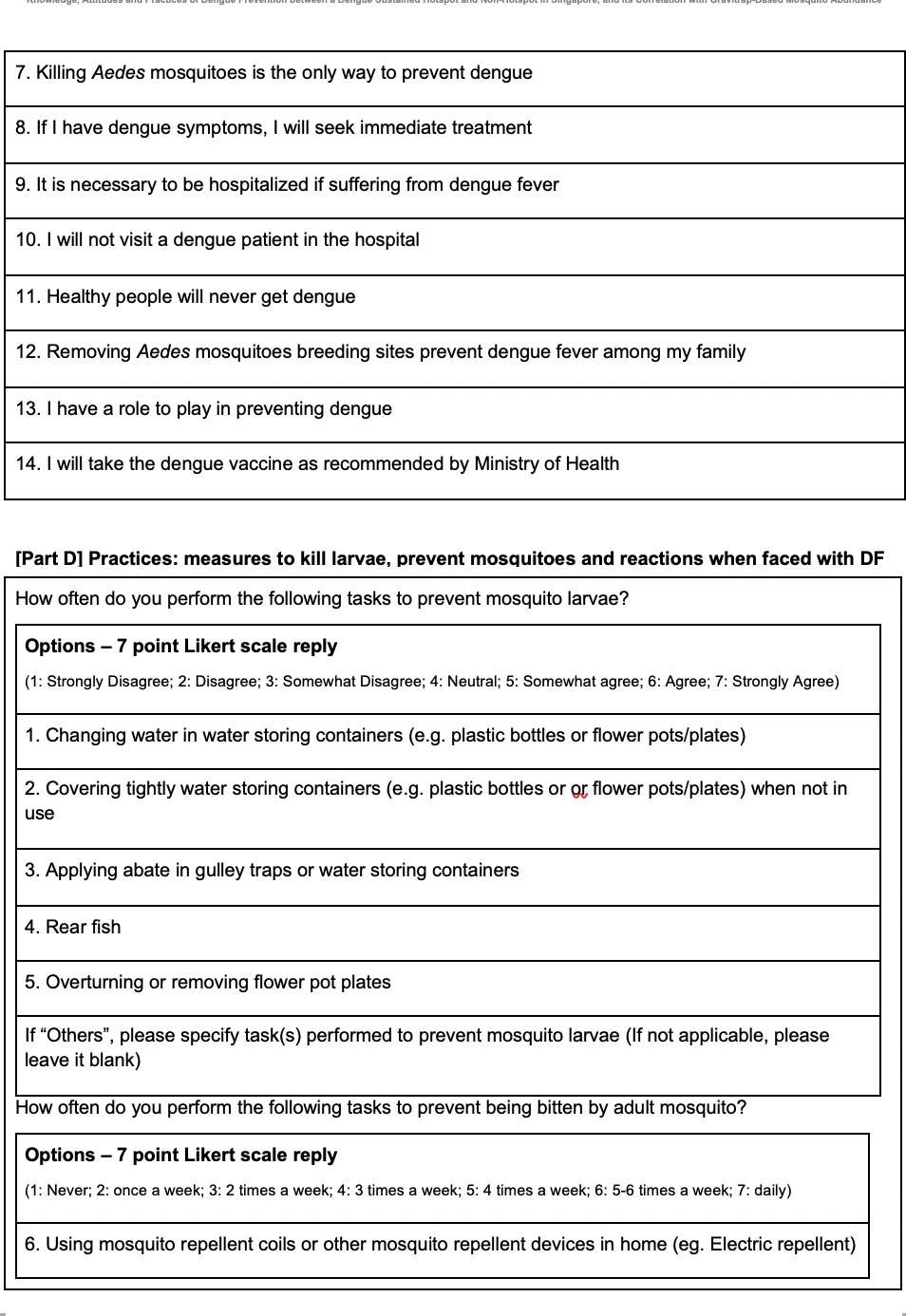
**

**
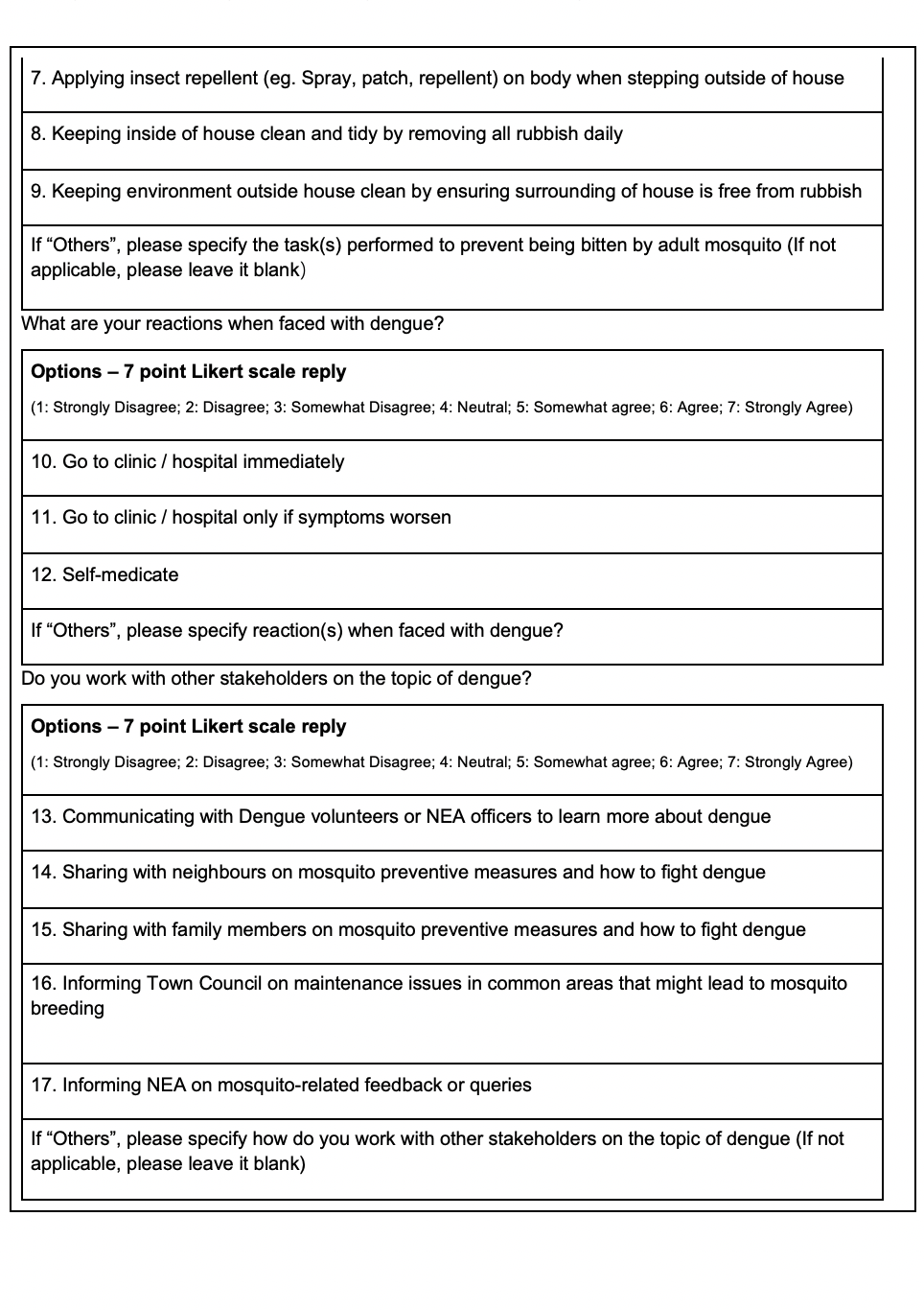
**

**Supplementary Figure 1.** KAP Questionnaire (English) [cont’d]


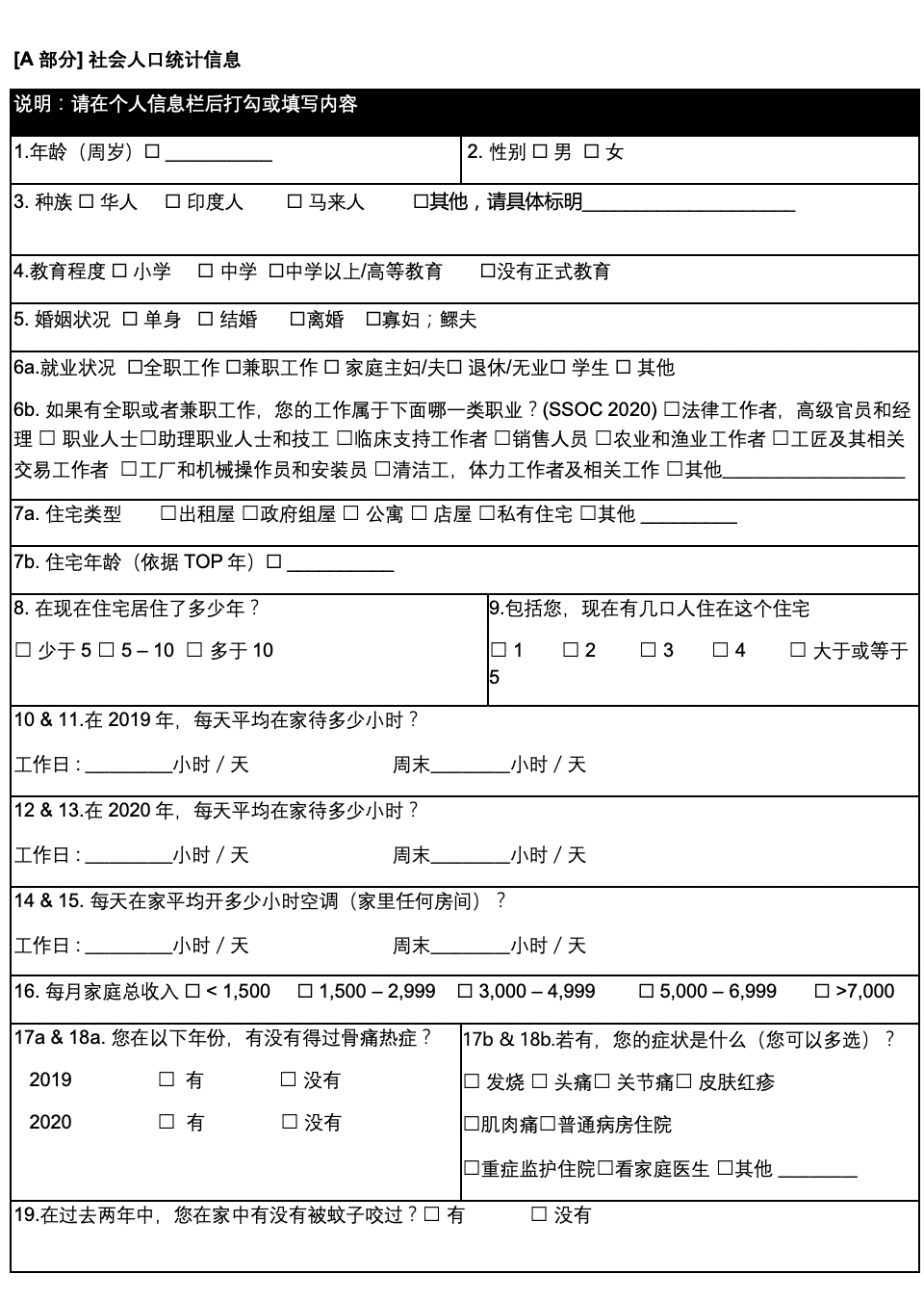

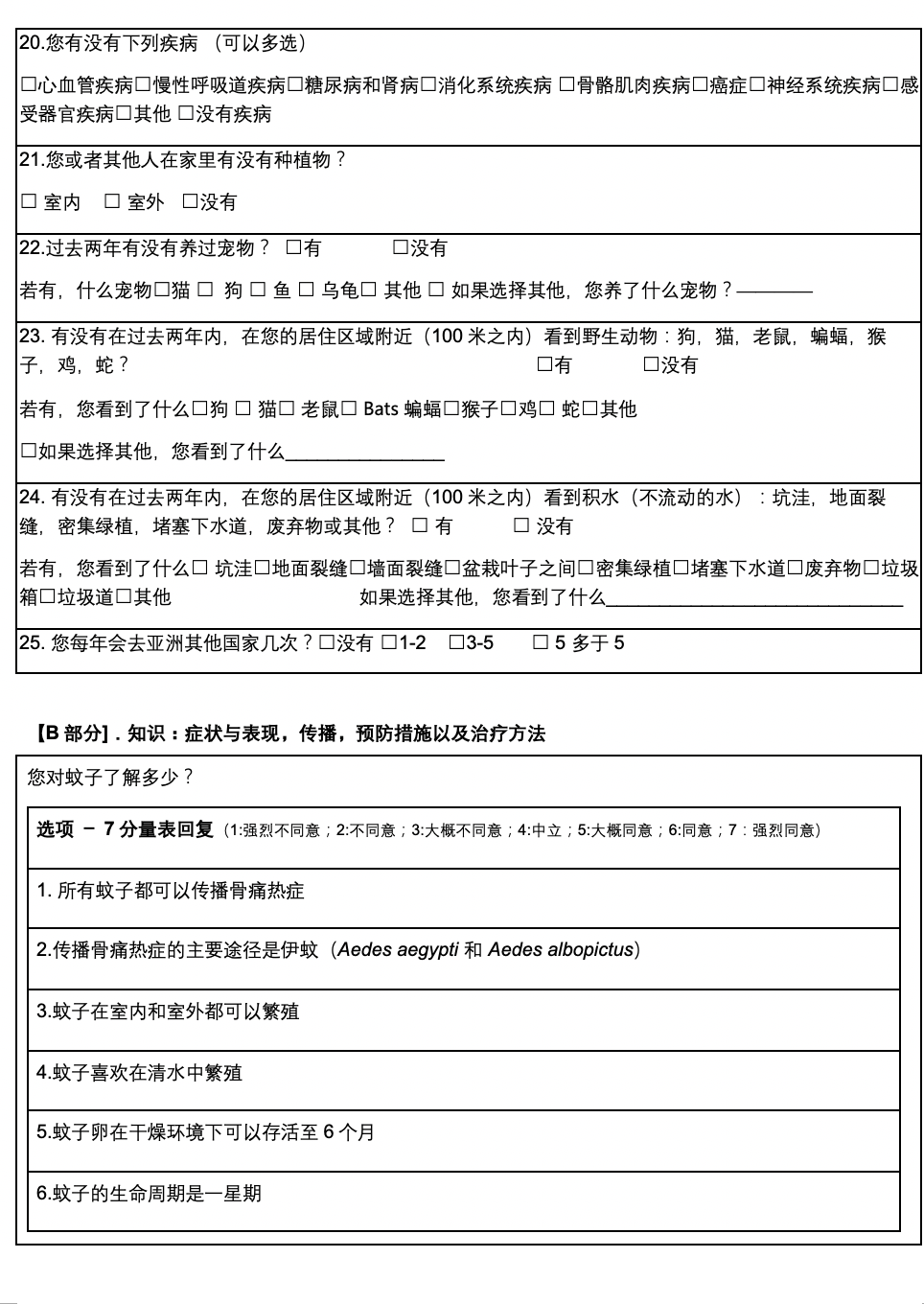


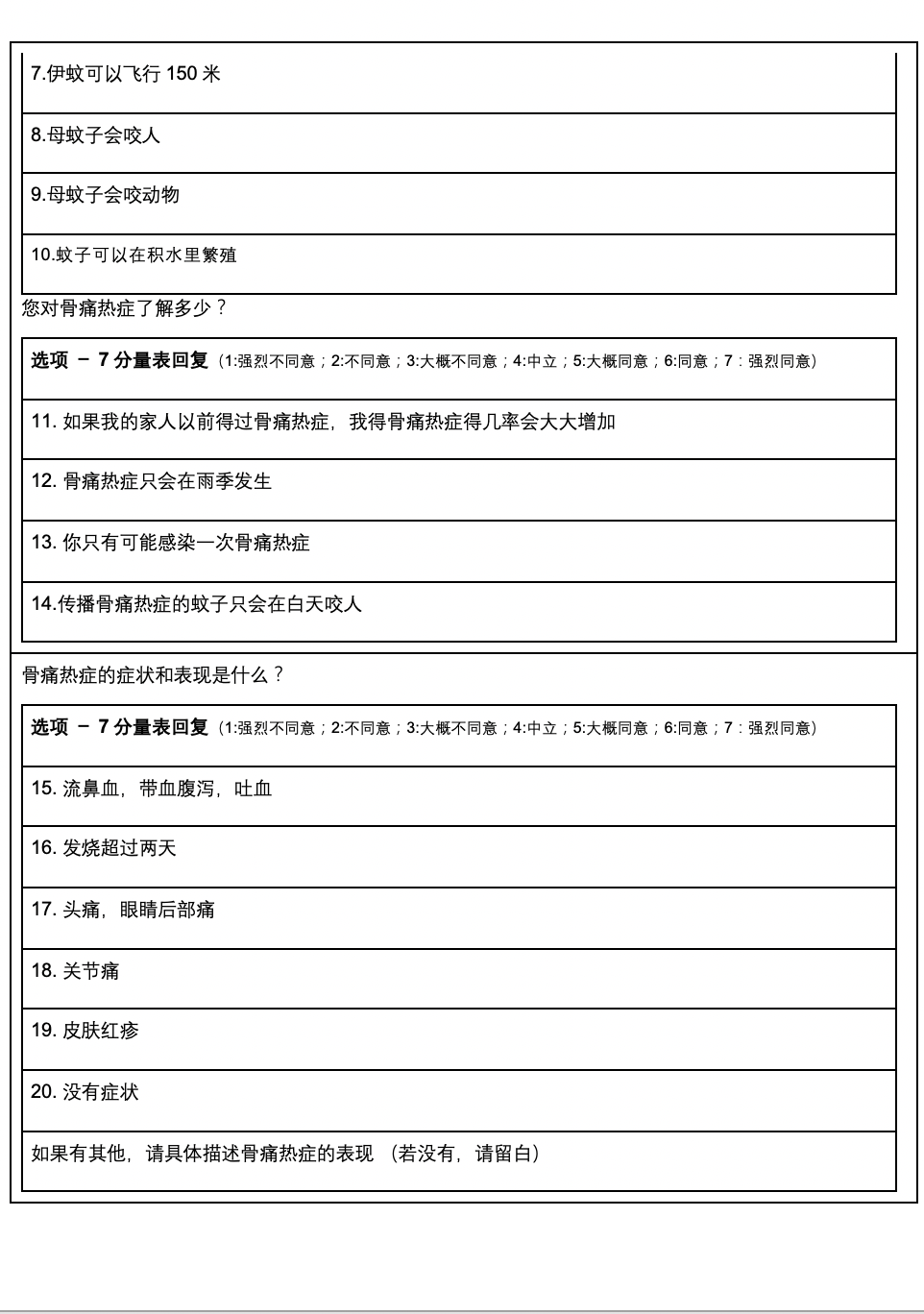

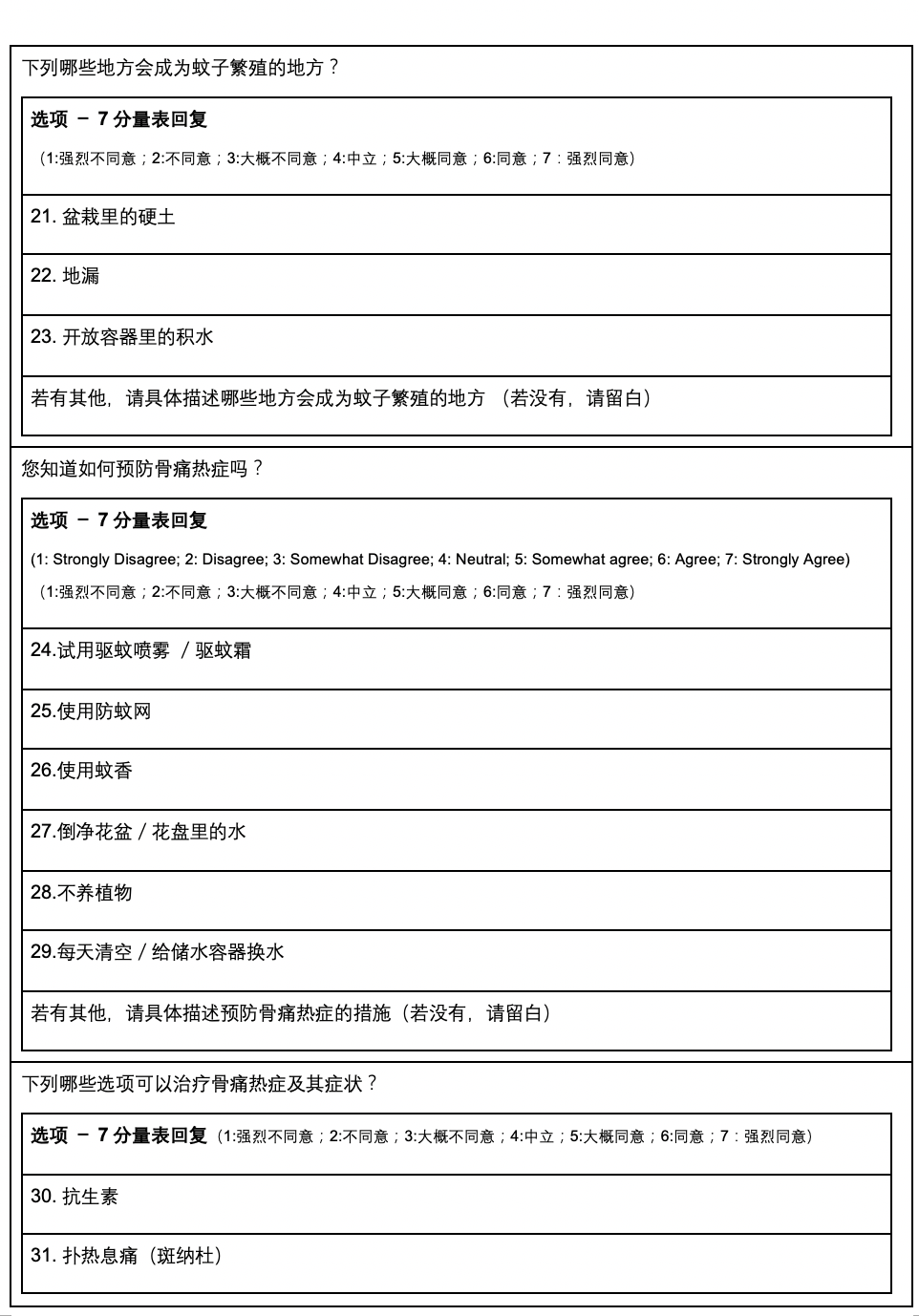


**Supplementary Figure 2.** KAP Questionnaire (Mandarin Translation)

**
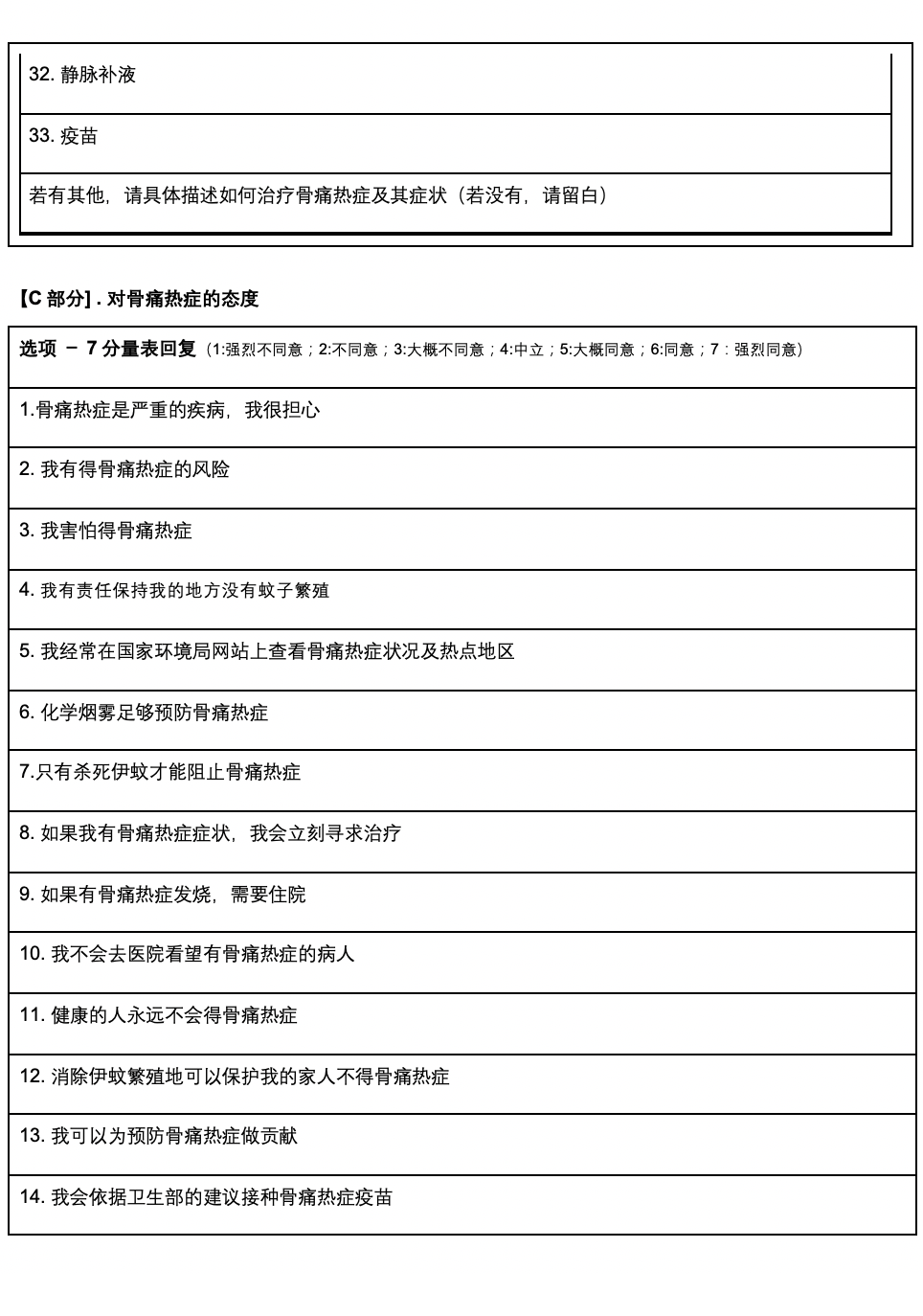

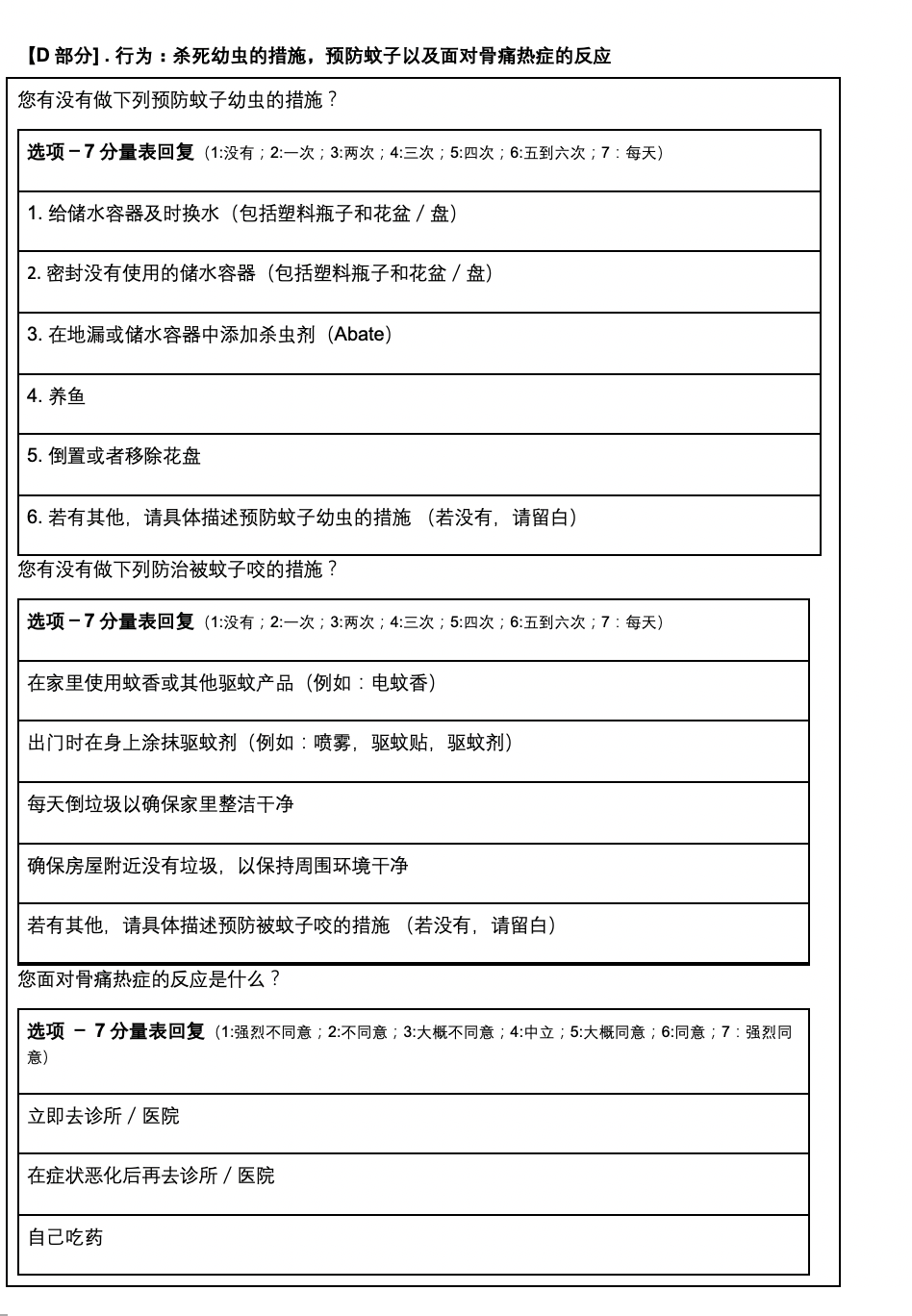
**

**
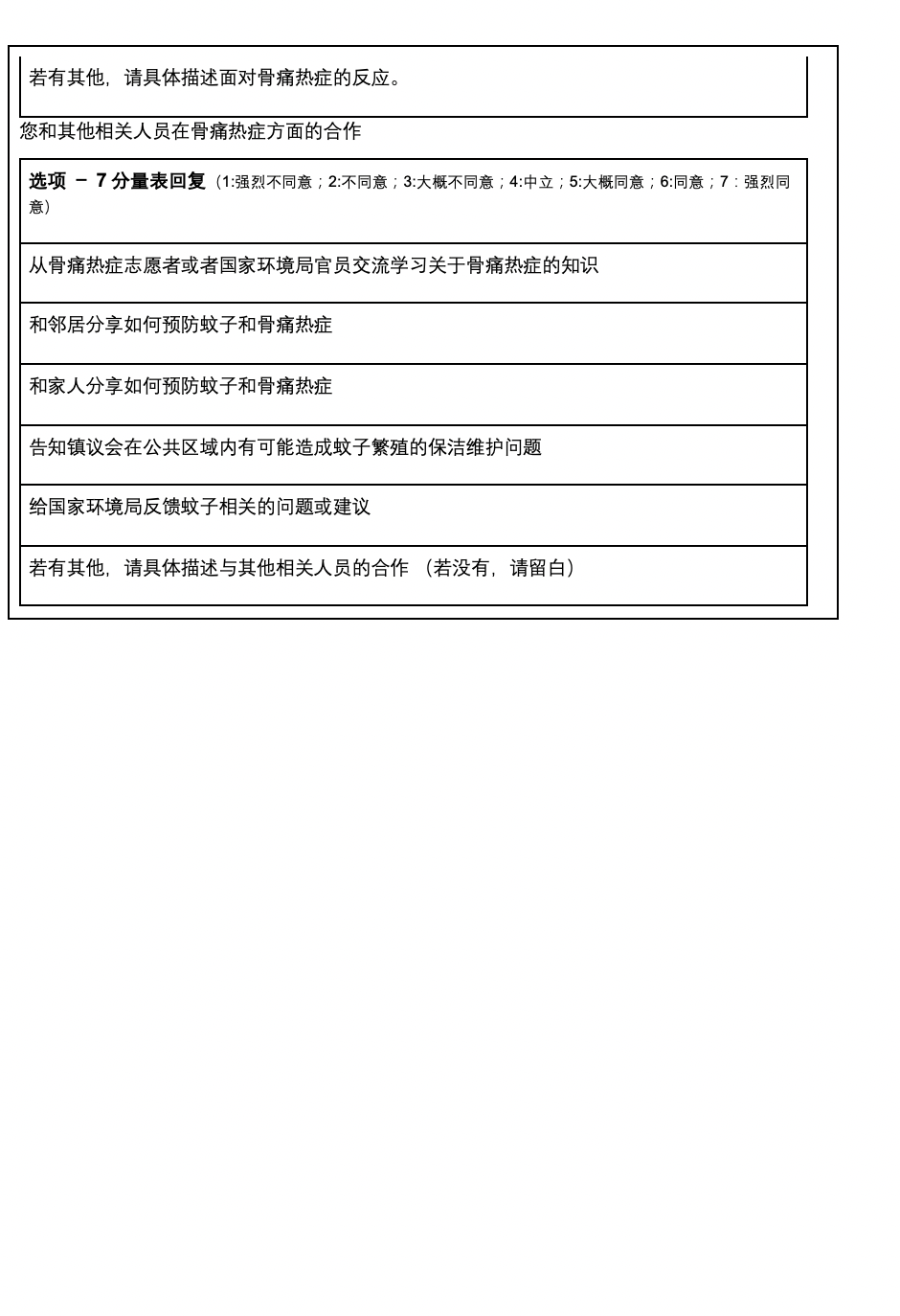
**

**Supplementary Figure 2.** KAP Questionnaire (Mandarin Translation) [cont’d]


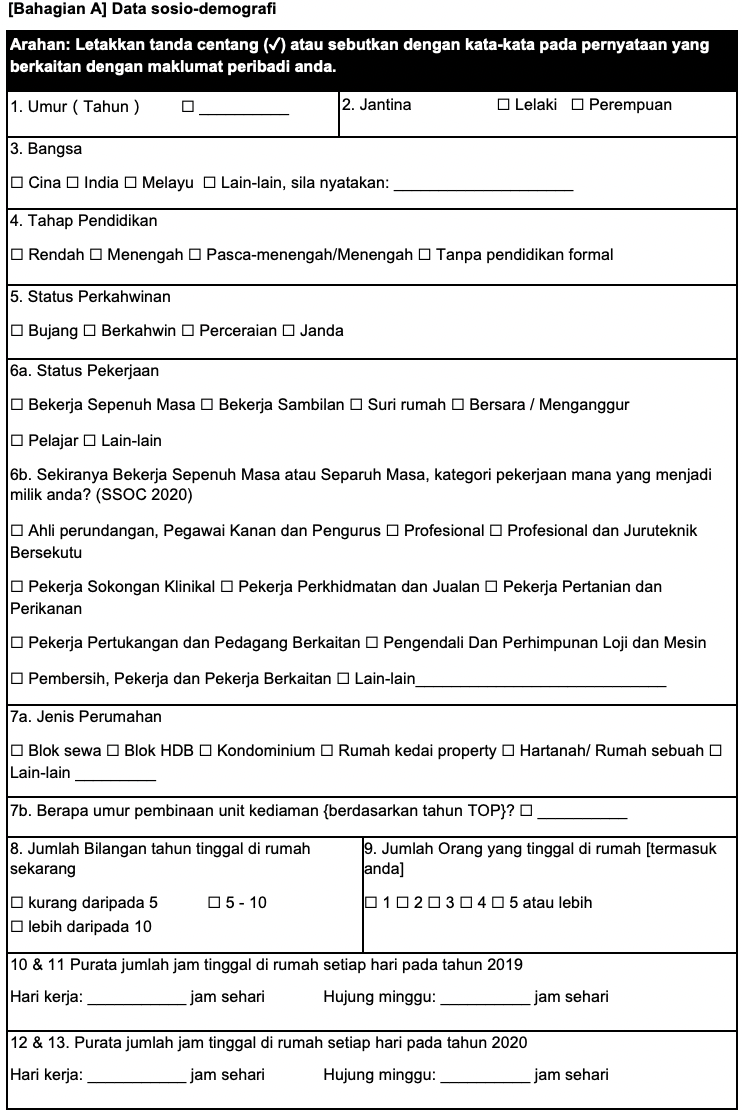

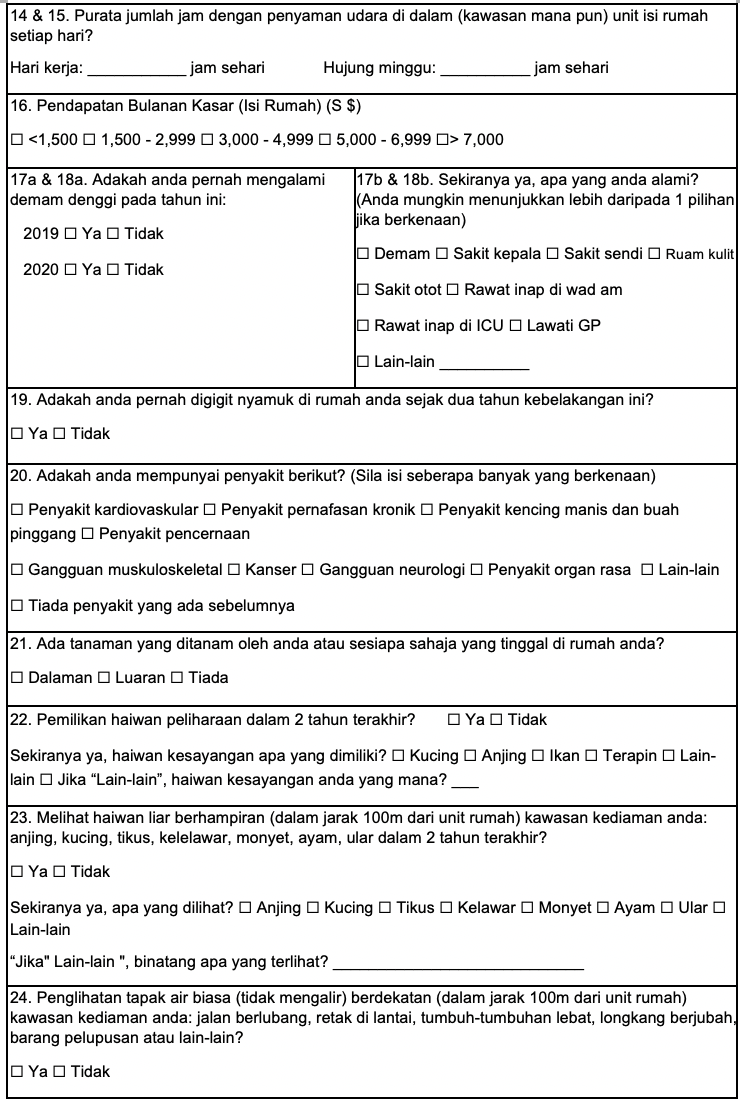


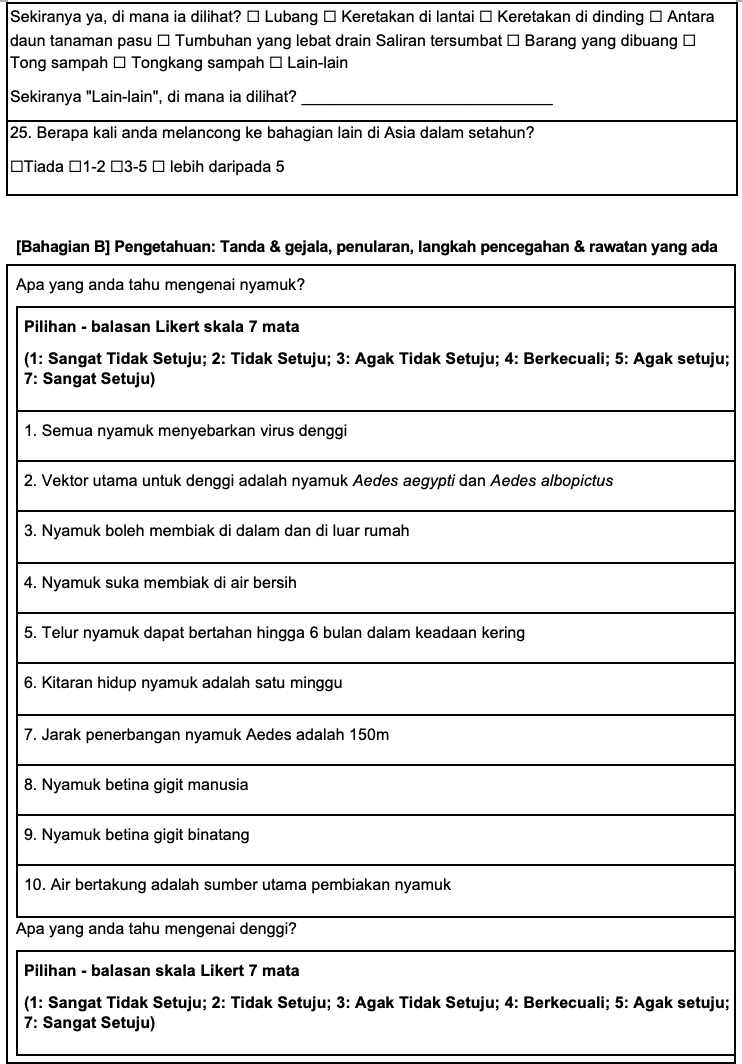

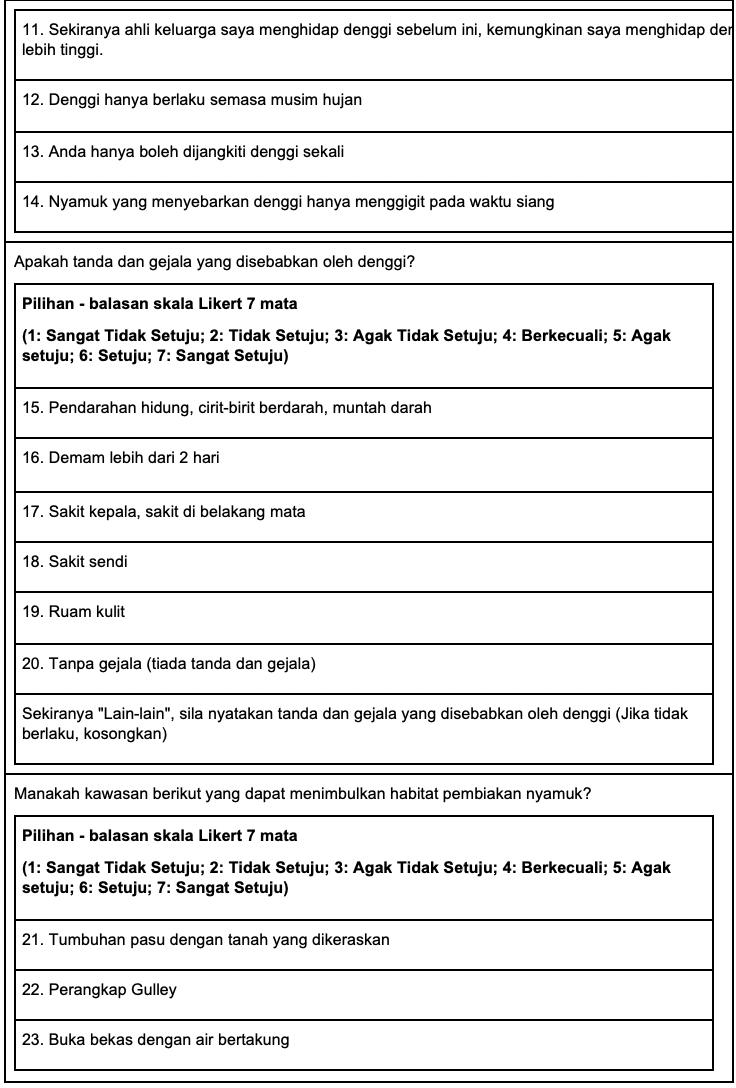


**Supplementary Figure 3.** KAP Questionnaire (Malay Translation)


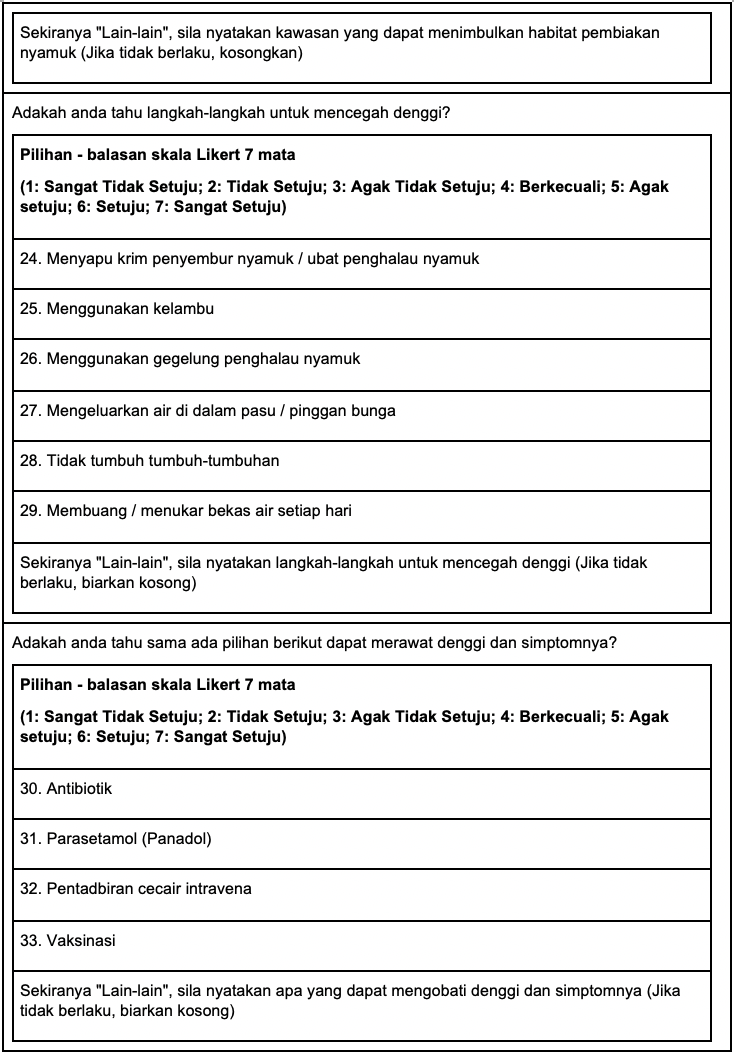

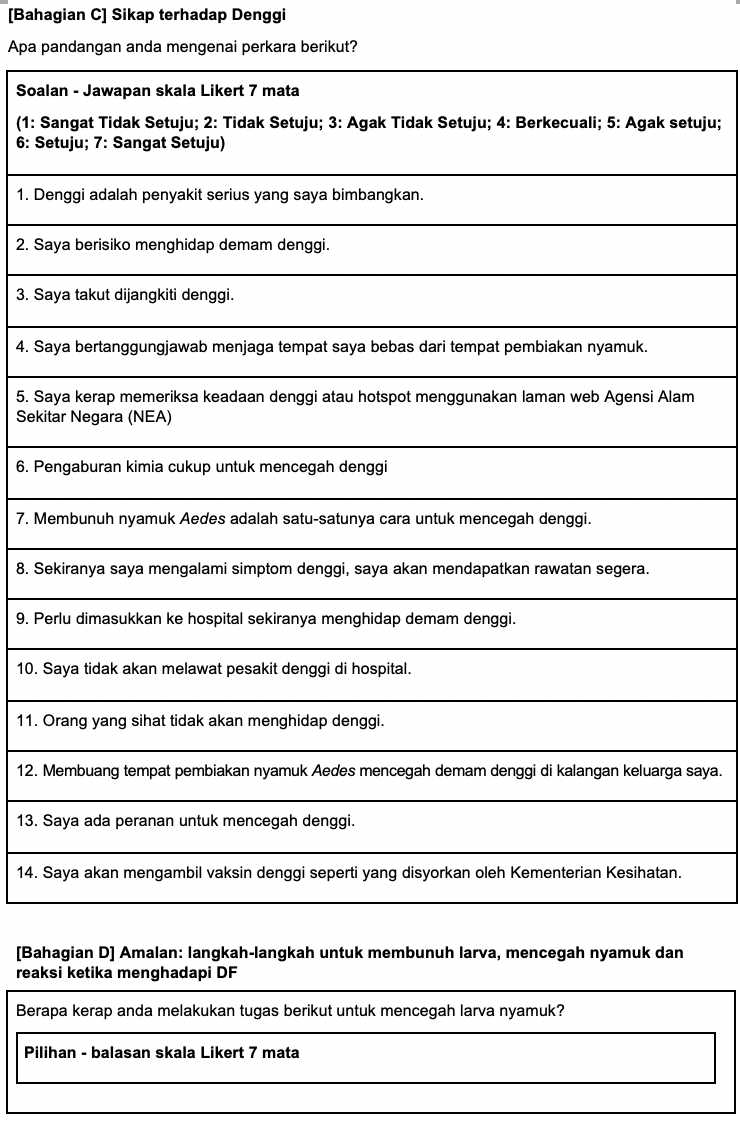


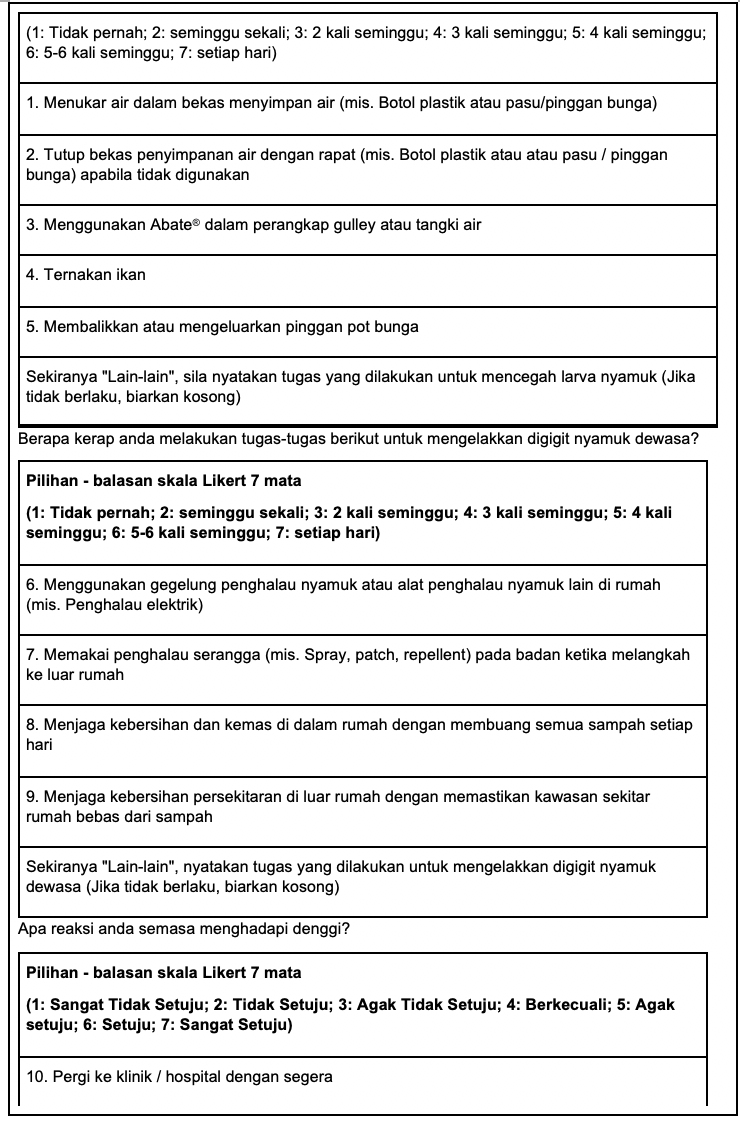

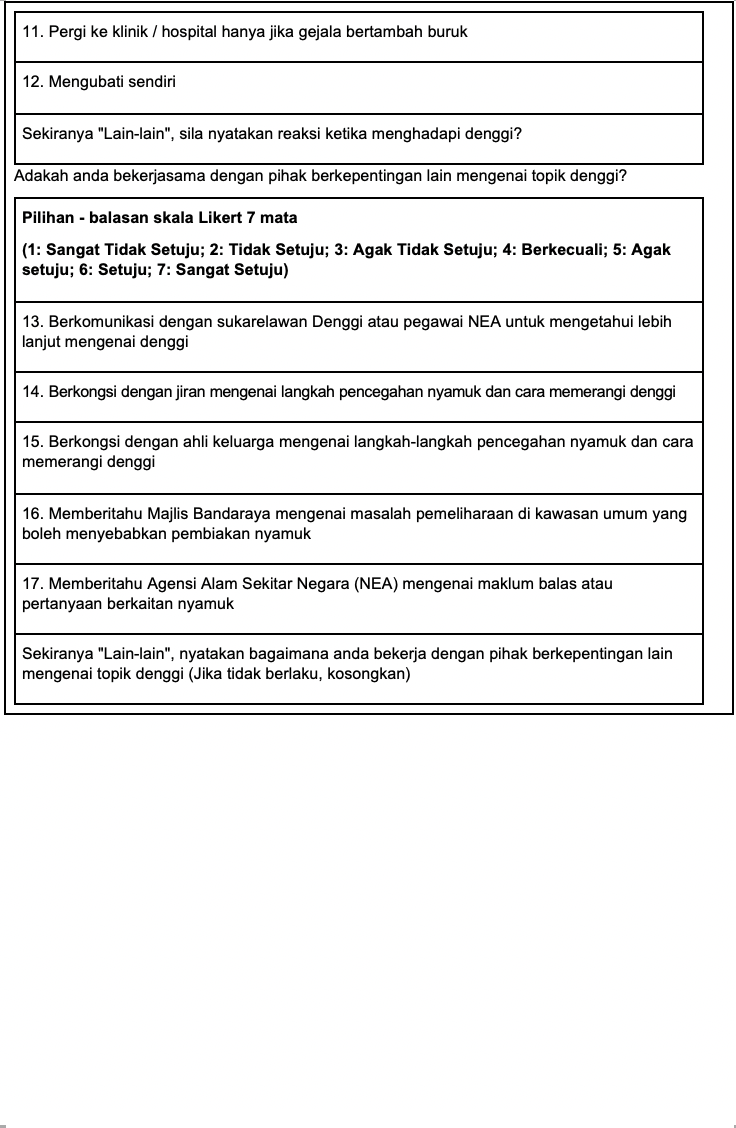


**Supplementary Figure 3.** KAP Questionnaire (Malay Translation) [cont’d]


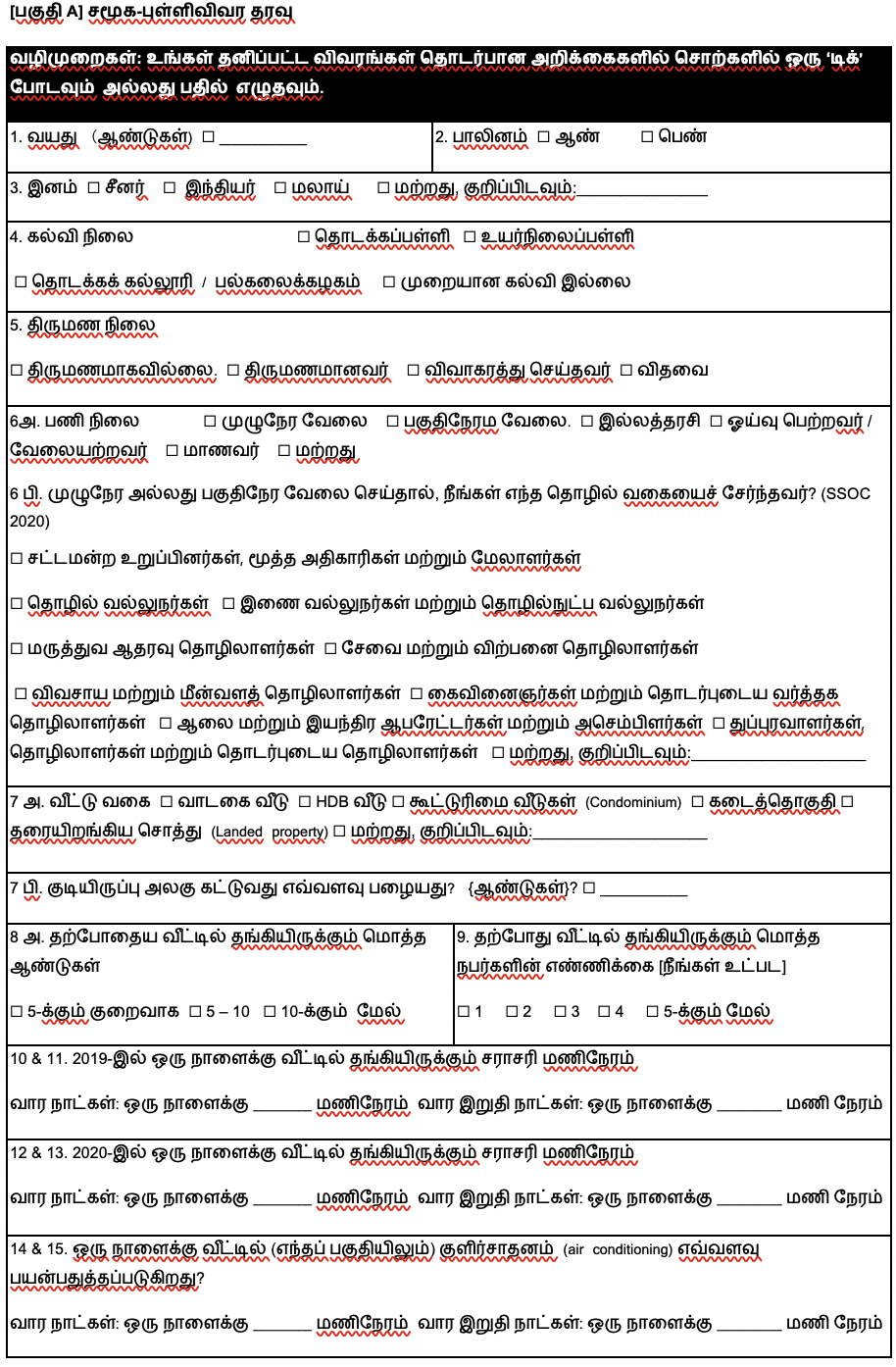

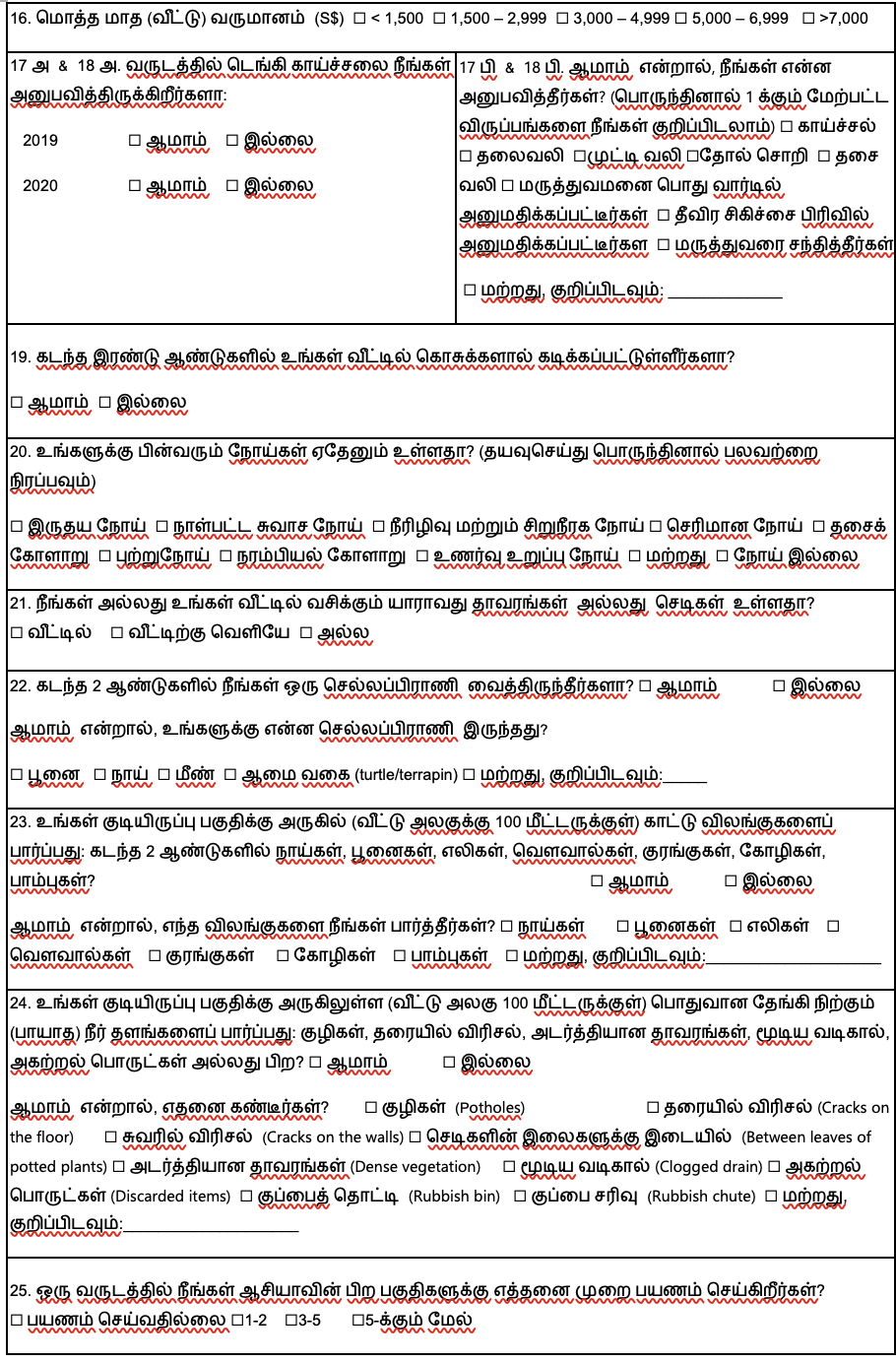


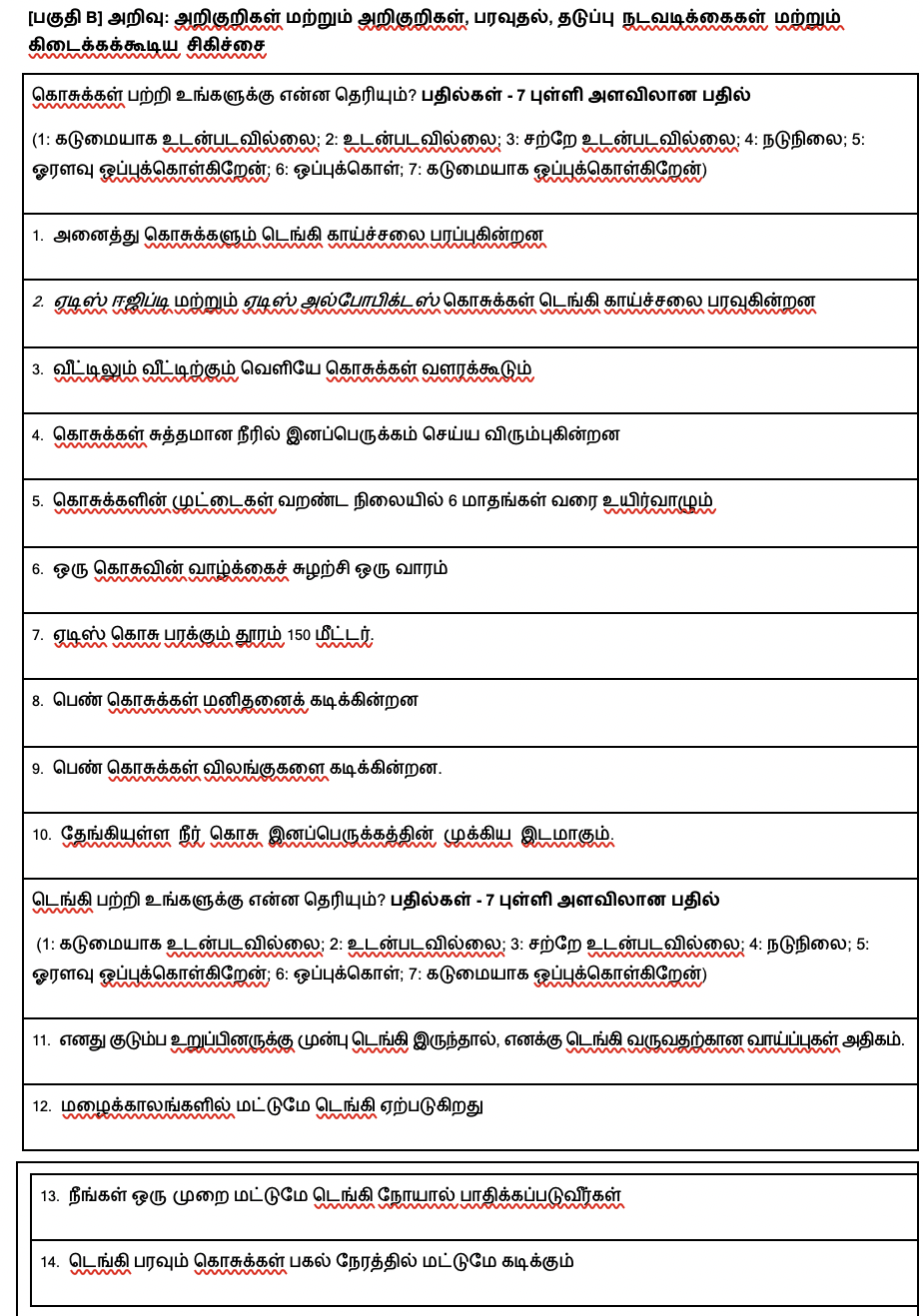

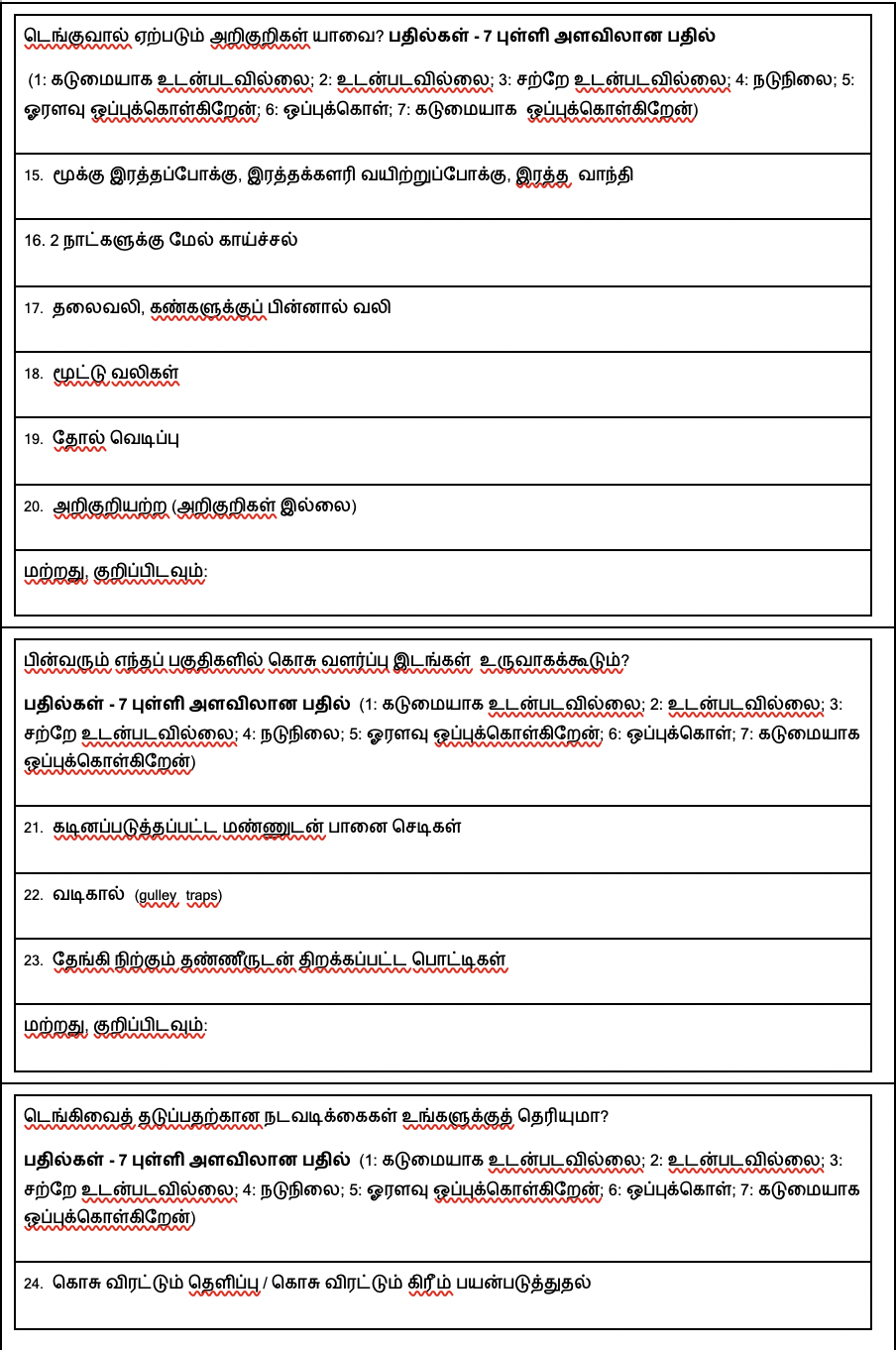


**Appendix Figure 4.** KAP Questionnaire (Tamil Translation)


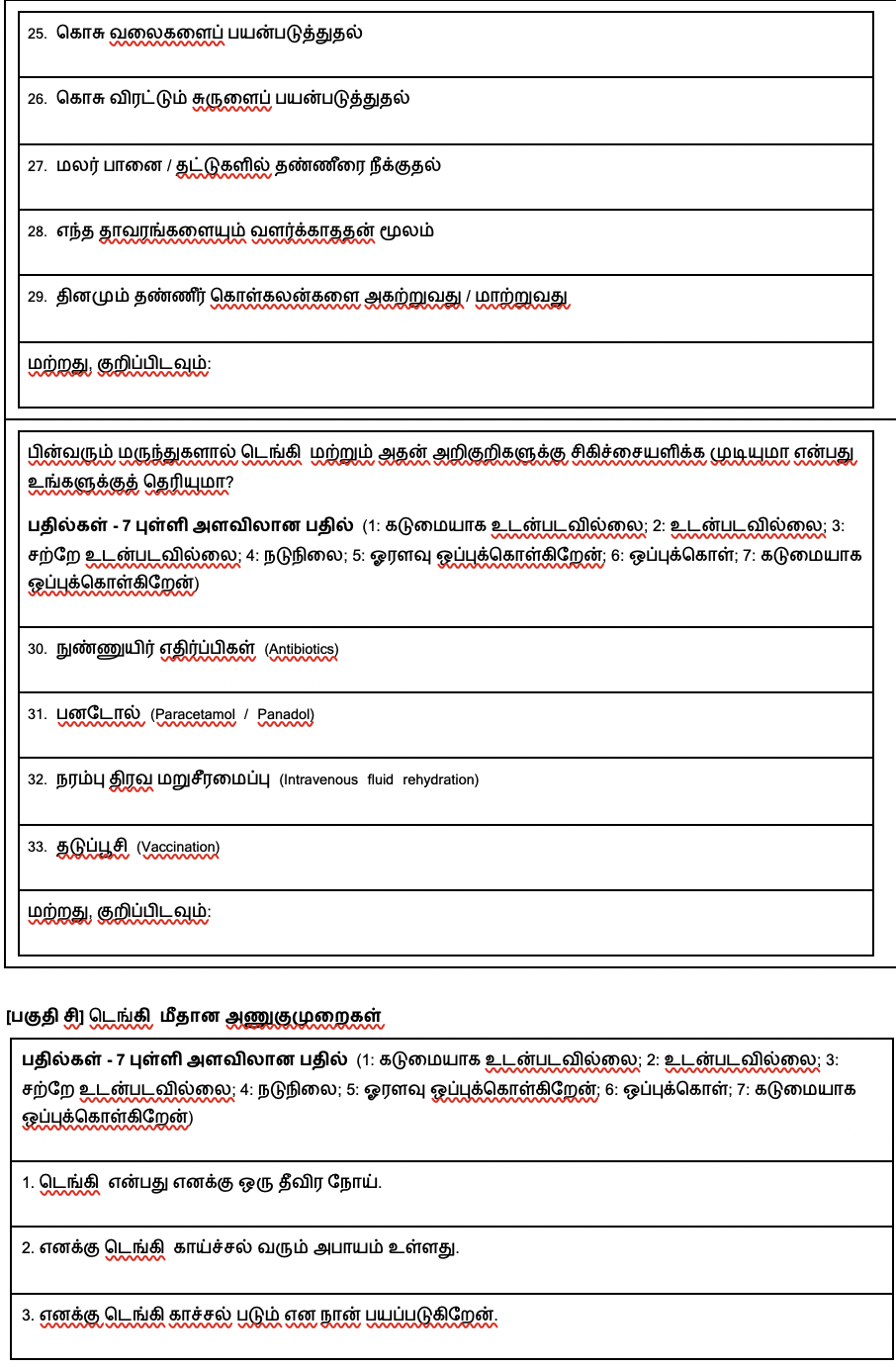

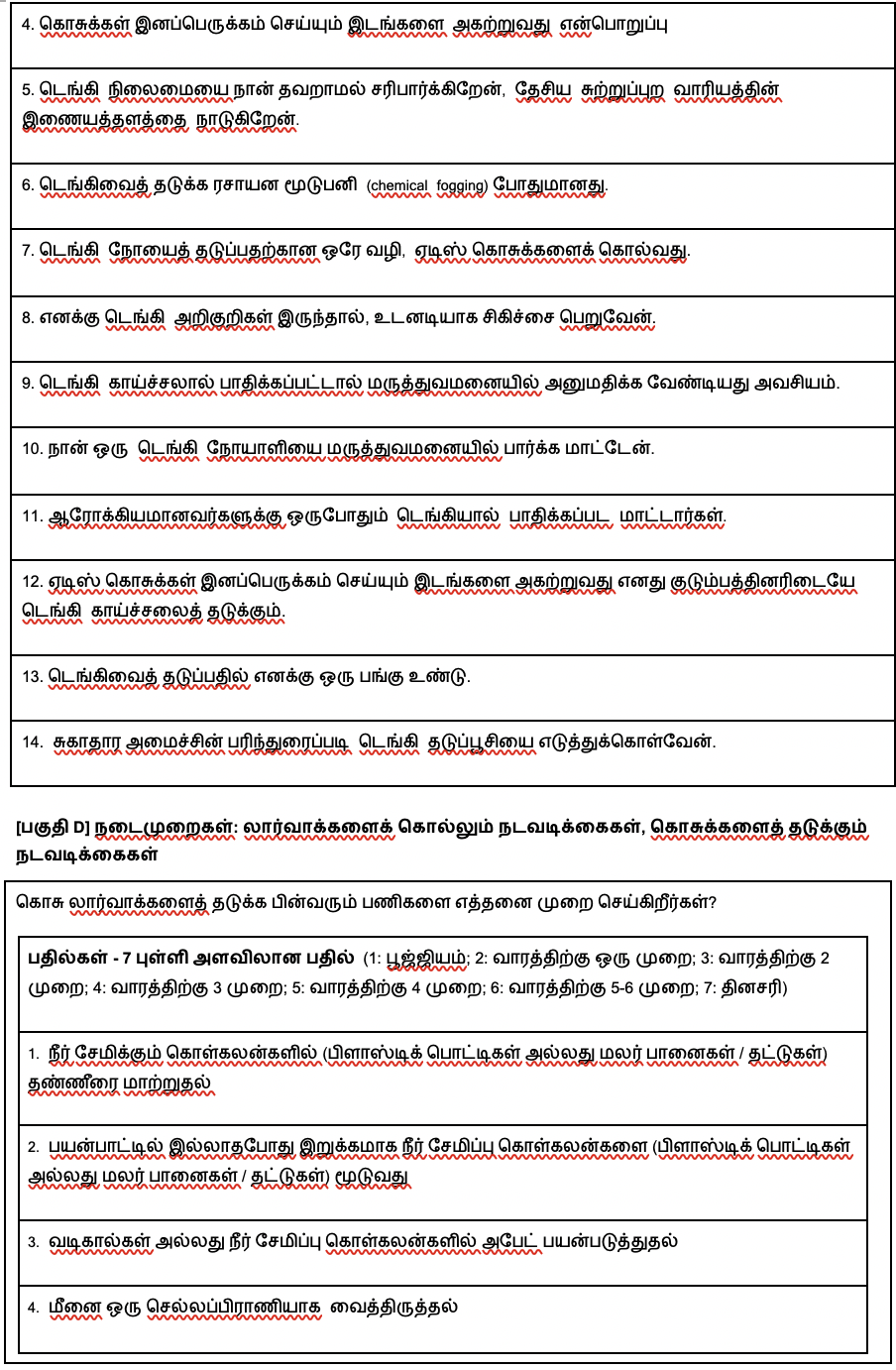


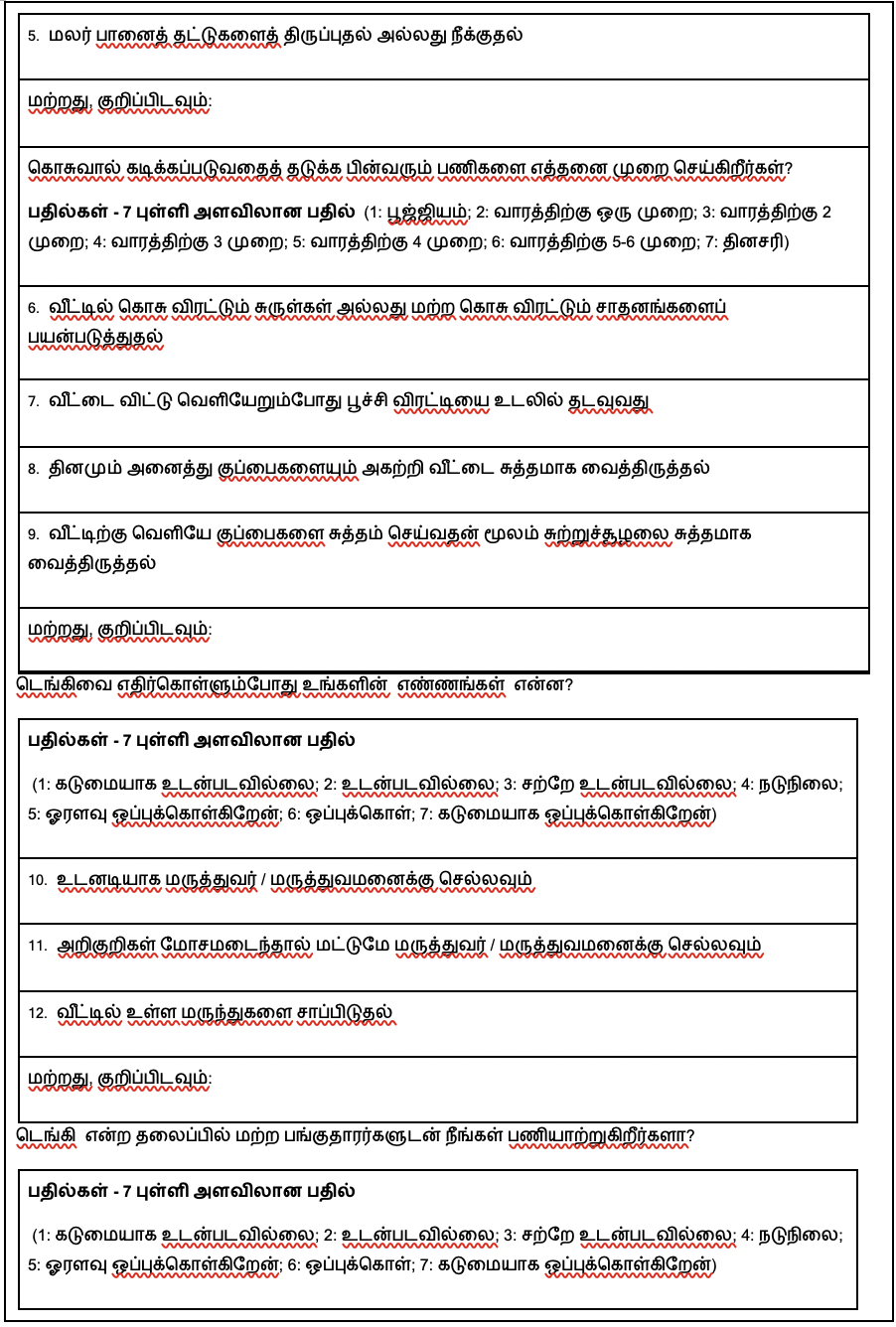

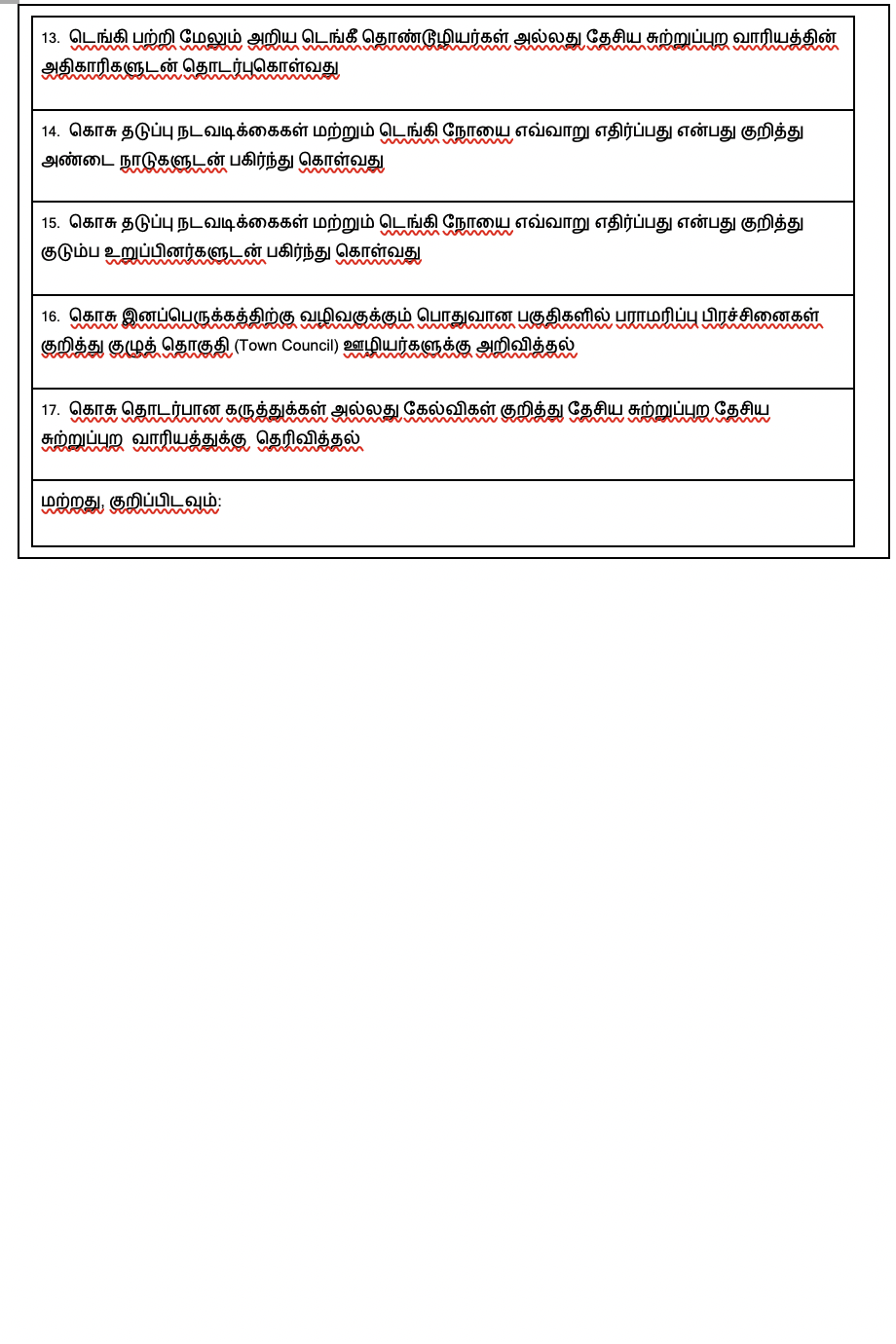


**Supplementary Figure 4.** KAP Questionnaire (Tamil Translation) [cont’d]

**
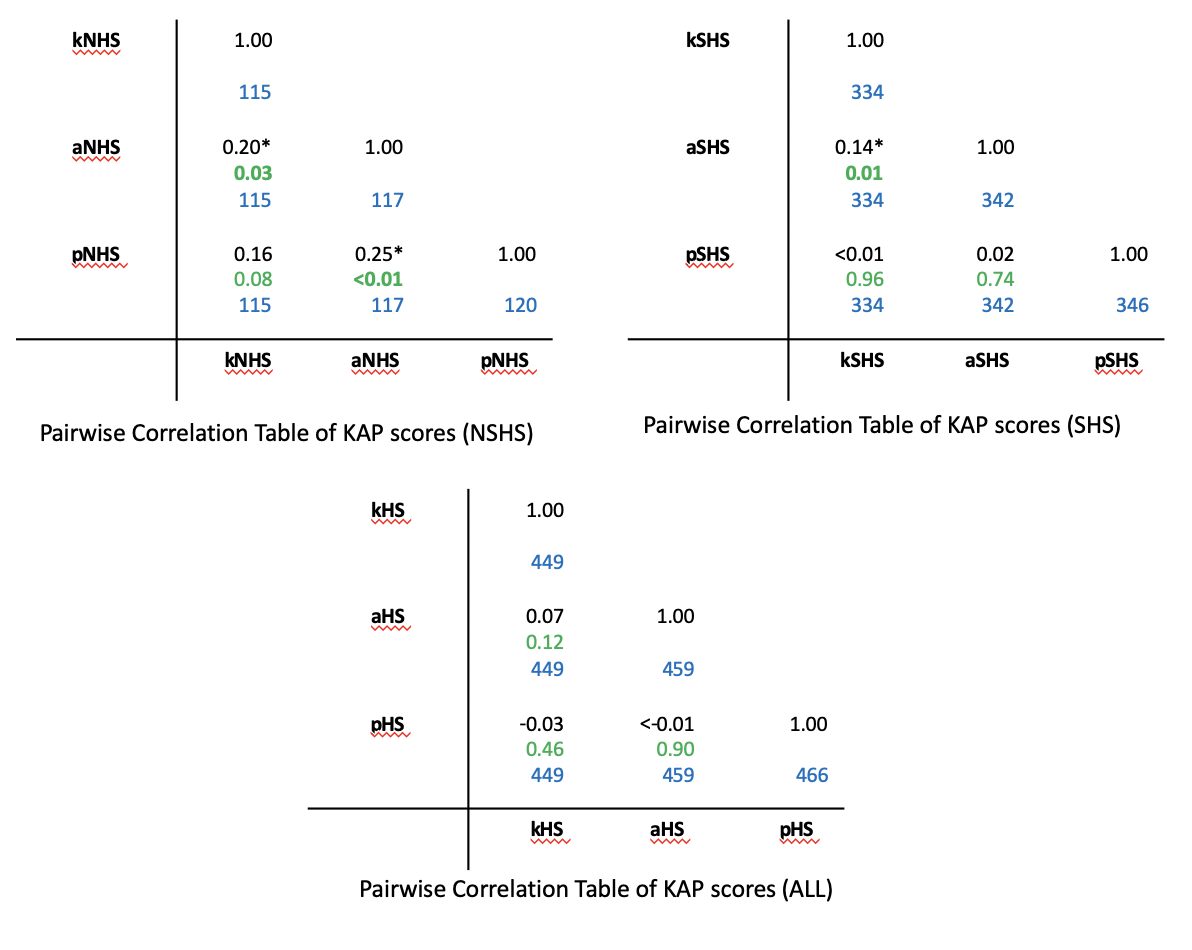
**

**Supplementary Figure 5.** Pairwise correlation table

**Note:** k: Knowledge; a: Attitudes; p: Practices; SHS: Sustained Hotspot; NSHS: Non Hotspot; HS (ALL): SHS +NHS; blue font: no. of observations; green font: significance level; *: p-value significant (<0.05)

**
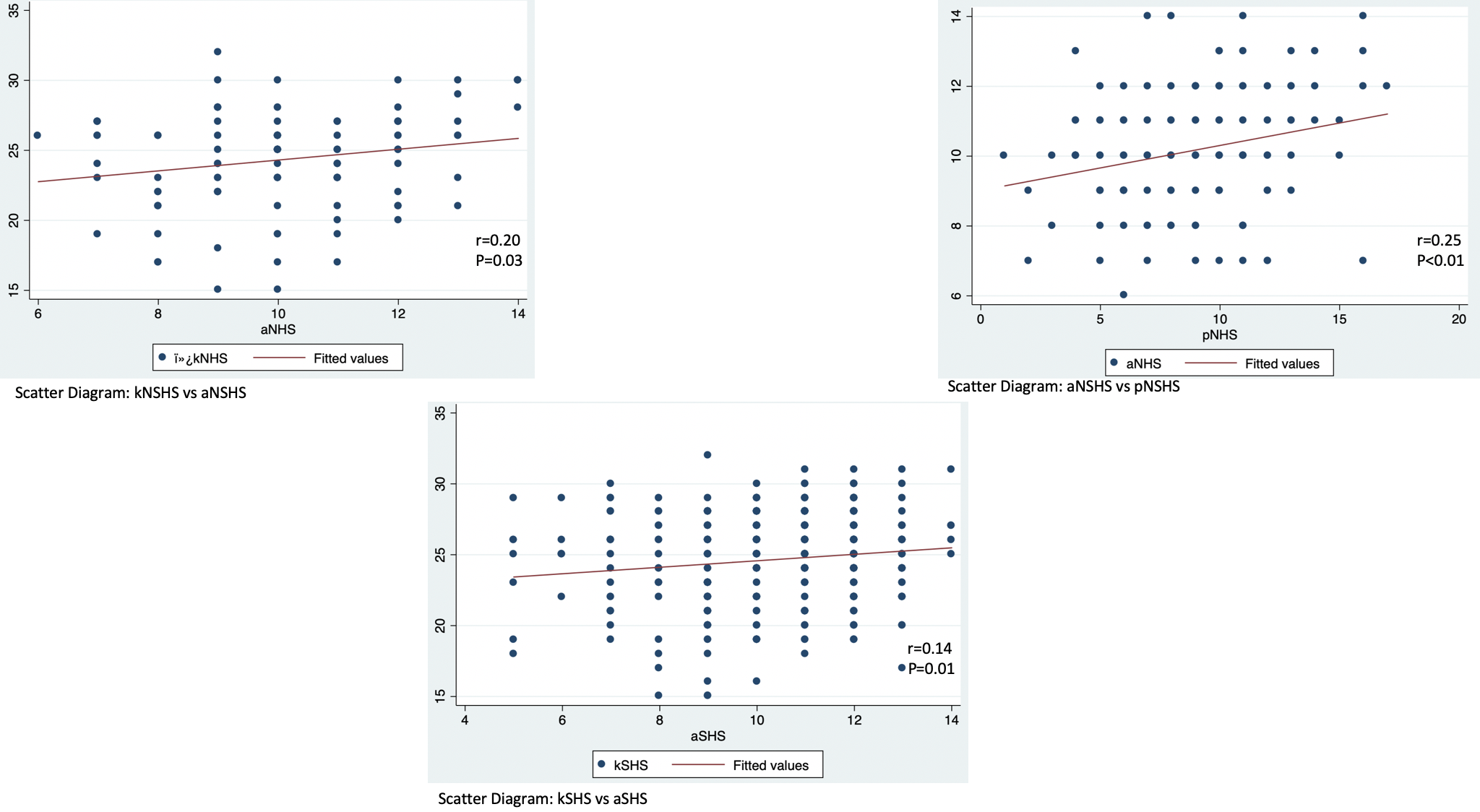
**

**Supplementary Figure 6.** Scatterplot of significant correlated K-A and A-P scores for NHS and SHS areas

**Note:** k: Knowledge; a: Attitudes; p: Practices; SHS: Sustained Hotspot; NSHS: Non-sustained Hotspot;

P-value significant (<0.05)


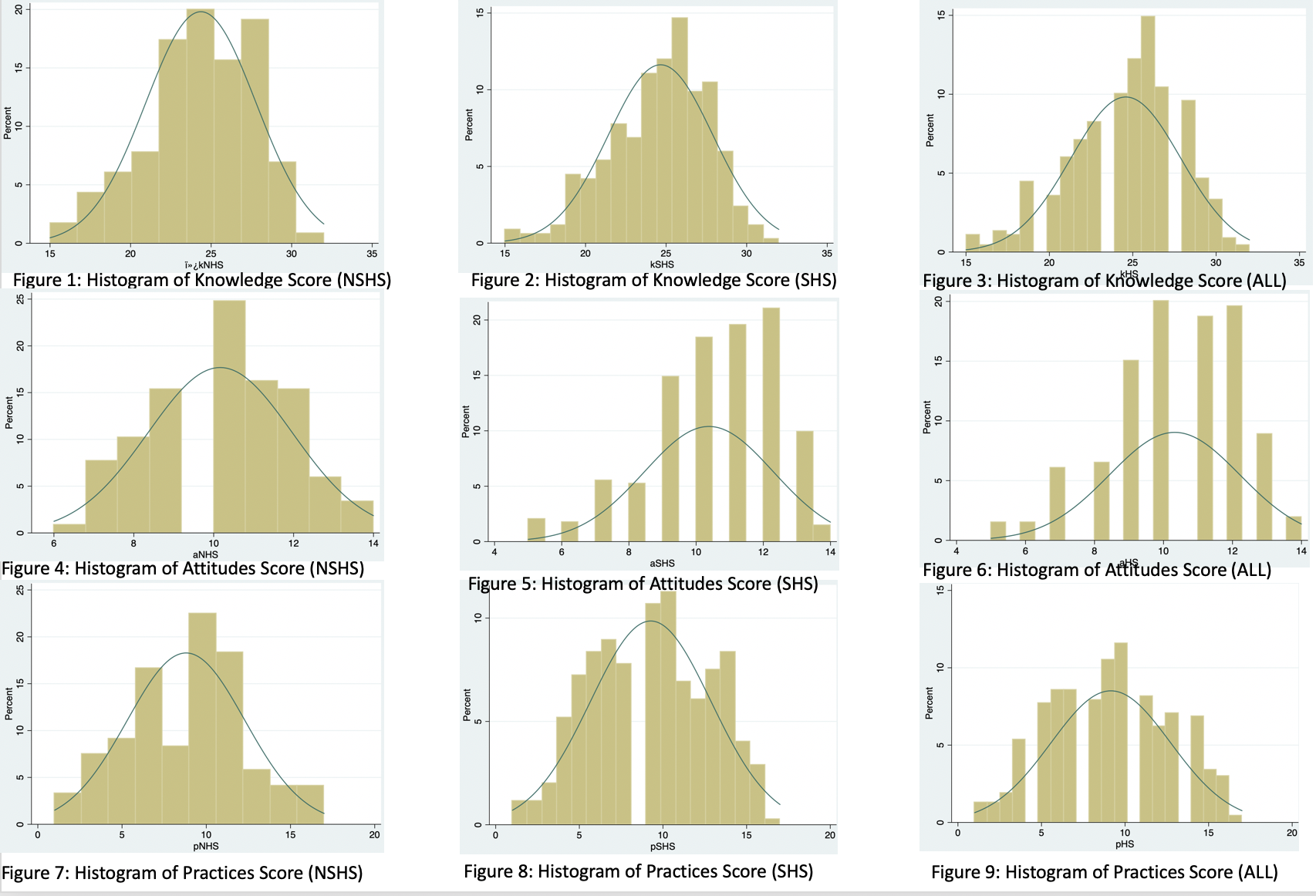


**Supplementary Figure 7.** Histogram depicting normal distribution of Knowledge, Attitudes and Practices Score in SHS, NSHS and HS areas

**Note:** k: Knowledge; a: Attitudes; p: Practices; SHS: Sustained Hotspot; NSHS: Non Hotspot; ALL: SHS +NHS; blue font: no. of observations; green font: significance level; *p-value significant (<0.05)


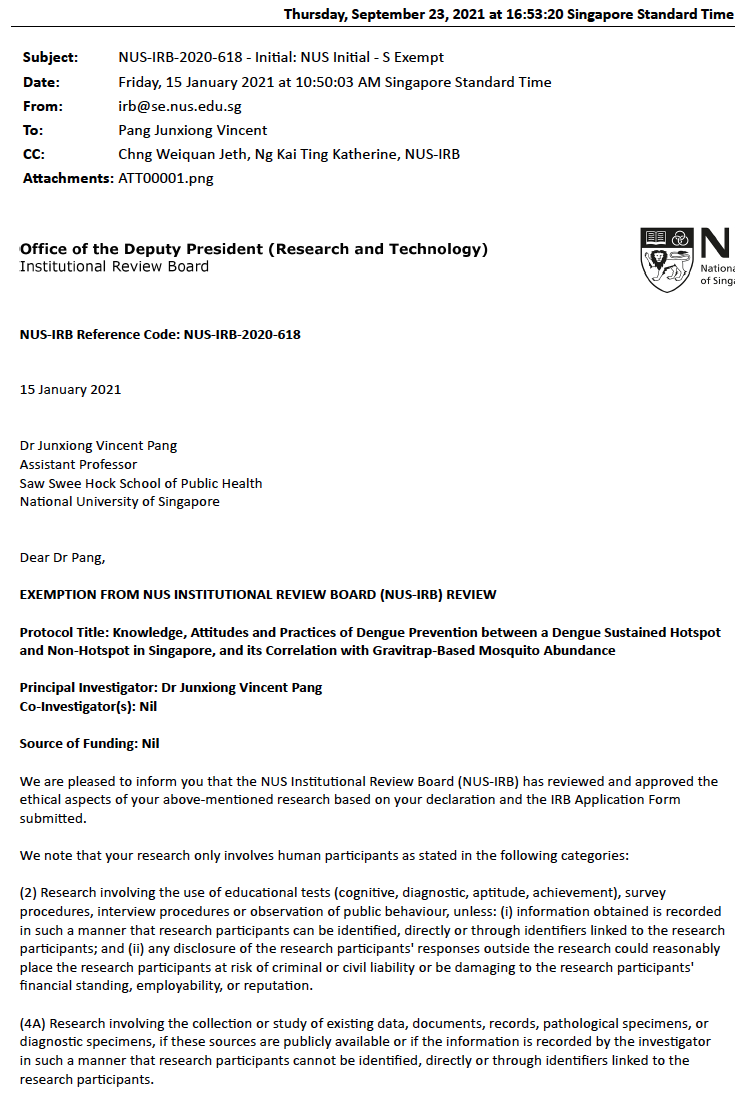

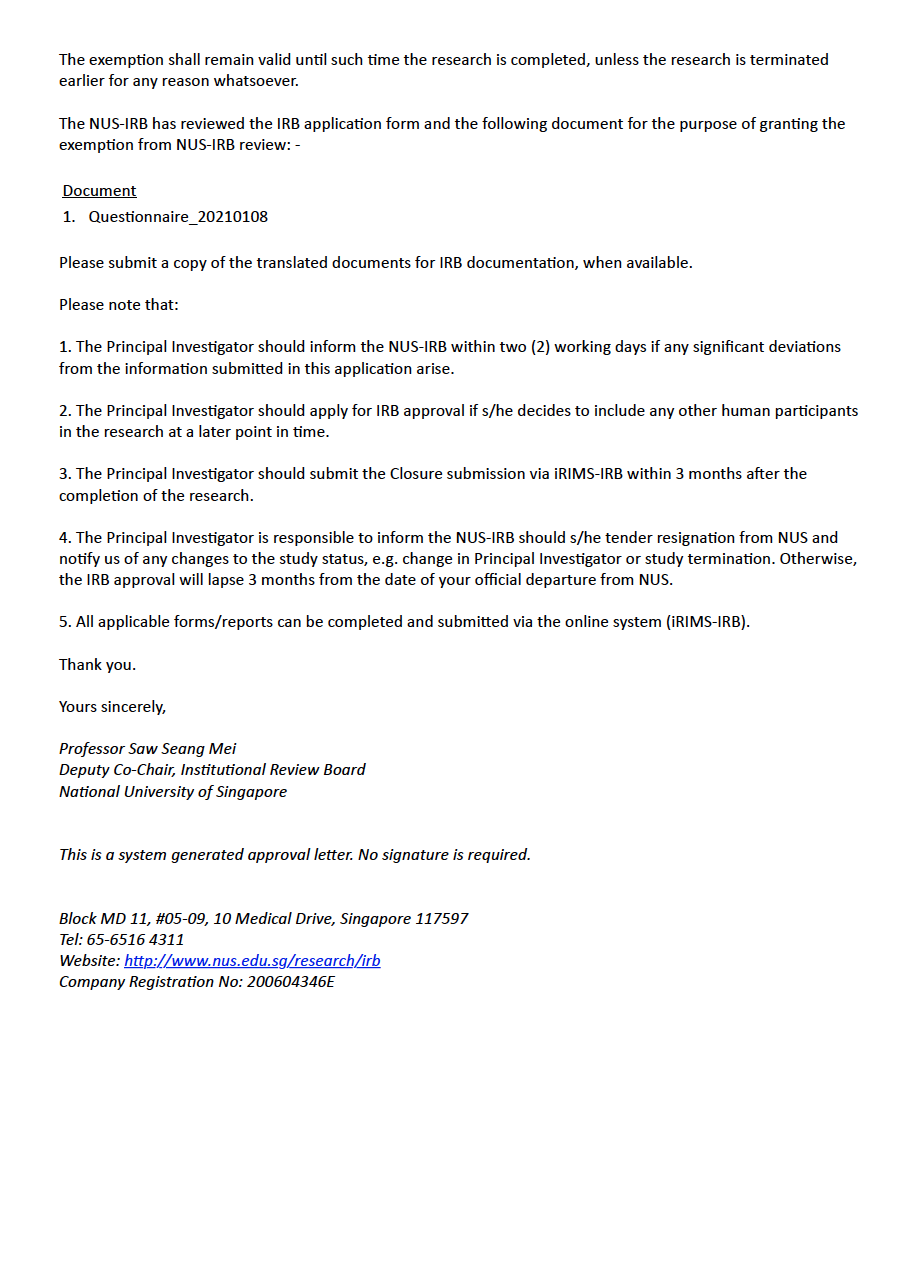


**Supplementary Figure 8**. NUS-IRB Reference Code: NUS-IRB-2020-618 - EXEMPTION FROM NUS INSTITUTIONAL REVIEW BOARD (NUS-IRB) REVIEW
